# An In Situ Embedded B‐MOF Sponge With Shape‐Memory for All‐in‐One Diabetic Wound Therapy

**DOI:** 10.1002/adhm.202505350

**Published:** 2026-01-22

**Authors:** Hai Zhou, Chaoyang Huang, Yanqi Chen, Tingzi Zhao, Fengyi Zhu, Gong Jun, Jinrong Zhang, Yingsong Pan, Qiulan Wen, Lei Yang, Huihui Zhang, Lianglong Chen

**Affiliations:** ^1^ Central Laboratory of Yunfu People's Hospital Yunfu People's Hospital Yunfu City P. R. China; ^2^ Department of Burns Nanfang Hospital Southern Medical University Guangdong P. R. China; ^3^ Department of Orthopaedic Surgery Nanfang Hospital Southern Medical University Guangzhou Guangdong P. R. China

**Keywords:** acellular dermal matrix, MOF, salvianolic acid B, wound healing

## Abstract

Diabetic wounds are characterized by persistent inflammation, impaired angiogenesis, and susceptibility to infection, posing significant clinical challenges. Here, we report an intelligent shape‐memory sponge (P_1_A_3_@B‐MOF) engineered for the programmable modulation of the diabetic wound microenvironment. The dressing consists of an interpenetrating network of oxidized pullulan and acellular dermal matrix, incorporating zeolitic imidazolate framework‐8 (ZIF‐8) metal–organic frameworks encapsulated with salvianolic acid B (SalB) via in situ self‐assembly. This design enables exudate‐triggered shape recovery and pH‐responsive drug release, targeting the acidic pathological environment. We demonstrate that the released Zn^2+^ and SalB exert synergistic effects: conferring broad‐spectrum antibacterial activity, orchestrating macrophage repolarization from proinflammatory M1 to regenerative M2 phenotypes, and activating the hypoxia‐inducible factor 1‐alpha (Hif‐1α)/vascular endothelial growth factor (VEGF) pathway to restore vascularization. In a diabetic rat model, the sponge accelerated wound closure with a 98.5% healing rate by day 14 and modulated collagen deposition via the transforming growth factor‐β (TGF‐β)/Smad signaling axis, thereby effectively mitigating scar formation. This integrated strategy offers a promising modality for restoring immune homeostasis and promoting functional skin regeneration.

## Graphical abstract

A smart shape‐memory sponge dressing (P_1_A_3_@B‐MOF) is developed for accelerated diabetic wound healing. It achieves pH‐responsive corelease of Zn^2^^+^ and salvianolic acid B, synergistically providing antibacterial action, repolarizing macrophages to the M2 phenotype, and promoting angiogenesis. In diabetic rats, it accelerated wound closure, regulated collagen via transforming growth factor‐β (TGF‐β)/Smad signaling, and reduced scarring.

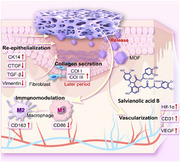

## 1 Introduction

Diabetic wounds represent one of the most severe complications of diabetes and are the leading cause of nontraumatic lower limb amputations, with severe cases leading to patient mortality [1]. Studies indicate that diabetes is the leading etiology of chronic wounds, followed by infection and pressure‐related injuries [2]. Therefore, developing comprehensive therapeutic modalities to simultaneously prevent and treat diabetes and diabetic wounds, thereby reducing mortality and disability rates and improving patients' quality of life, is a major public health priority [3]. The development of diabetic wounds results from the combined effects of impaired endogenous healing mechanisms and exogenous environmental factors, with endogenous factors playing a decisive role [4]. Notably, persistent hyperglycemia, chronic inflammation, extensive neurovascular complications, and tissue ischemia and hypoxia resulting from vascular impairment exacerbate the disturbance of immune homeostasis and impede wound healing in patients with diabetes [5]. This leads to the disruption of the normal wound healing cascade, resulting in prolonged inflammation, arrested angiogenesis during the inflammatory and proliferative phases, abnormal granulation tissue formation, and ultimately, nonhealing wounds [6]. Consequently, strategies that remodel redox and immune homeostasis, restore vascular networks, and activate reparative cellular functions, may serve as promising strategies to accelerate diabetic wound healing [7].

Salvianolic acid B (SalB) is one of the most abundant water‐soluble components of *Salvia miltiorrhiza* and a primary active ingredient responsible for its pharmacological effects [8]. Possessing abundant phenolic hydroxyl groups, SalB exhibits potent antioxidant potential. Research has shown that SalB possesses a range of pharmacological properties, including antioxidant, anti‐inflammatory, and vascular endothelial protective effects [9]. Its antioxidant activity is achieved by scavenging free radicals, inhibiting lipid peroxidation, and enhancing endogenous antioxidant enzyme activity. Furthermore, its anti‐inflammatory effects are mediated by the suppression of inflammatory signaling pathways, such as nuclear factor kappa‐B (NF‐κB), thereby reducing the production of proinflammatory cytokines [10]. These broad pharmacological activities provide a solid foundation for the application of SalB in wound repair. SalB upregulates the expression of multiple proangiogenic factors, including vascular endothelial growth factor (VEGF), basic fibroblast growth factor (bFGF), and angiopoietin‐1 (Ang‐1) [11]. VEGF is one of the most critical regulators of angiogenesis, and SalB promotes its expression and secretion by activating the Hif‐1α/VEGF signaling pathway. Additionally, SalB downregulates angiogenesis inhibitors, such as endostatin and angiostatin, creating a microenvironment conducive to vascularization [12]. The proangiogenic effect of SalB involves the activation of multiple signaling pathways. Studies have shown that SalB promotes nitric oxide (NO) production by activating the PI3K/Akt pathway, thereby improving endothelial cell function [13]. It also activates the Wnt/β‐catenin pathway, upregulating the expression and nuclear translocation of β‐catenin, which in turn promotes the transcription of angiogenesis‐related genes [14]. Collectively, these pathways underpin the provascularization potential of SalB, rendering it a highly promising angiogenic agent for tissue repair strategies. However, the clinical utility of SalB faces significant hurdles. Despite its high water solubility, the therapeutic efficacy of SalB is compromised by rapid in vivo metabolism, a short half‐life, and low bioavailability, making it difficult to maintain effective therapeutic concentrations locally at the wound site [15]. Conventional topical delivery systems, such as plain gels or creams, offer limited benefits owing to uncontrolled drug release and short residence time. Consequently, various delivery strategies have been explored to overcome these limitations. For instance, natural polymer‐based hydrogels, including Collagen sponge and hyaluronic acid, have been utilized as carriers for SalB. While retention is improved, these carriers typically lack intelligent stimuli‐responsive capabilities. Furthermore, while certain liposomal or polymeric nanoparticles allow sustained release, they remain challenged by limited drug‐loading capacity and stability issues.

Compared to conventional systems, metal–organic frameworks (MOFs) exhibit unique advantages in high‐efficiency drug loading and controlled release owing to their extremely high specific surface area and tunable pore structures [16]. Notably, zeolitic imidazolate framework‐8 (ZIF‐8), a typical MOF, has attracted considerable attention owing to its mild synthesis conditions, good biocompatibility, and unique pH‐responsive degradation behavior in acidic microenvironments [17]. ZIF‐8 is composed of zinc ions (Zn^2+^) and 2‐methylimidazole ligands, self‐assembling into a porous crystalline material with high specific surface area, adjustable pore size, and excellent chemical stability [18]. Its structure resembles that of sodalite (SOD), with a small pore aperture of 3.4 Å and a large cavity of 11.6 Å [19]. ZIF‐8 has been used in CO_2_ capture, H_2_ storage, and hydrocarbon separation due to its ability to selectively adsorb small gas molecules. It also serves as a catalyst support and participates directly in catalytic reactions, such as CO_2_ hydrogenation to methanol and degradation of organic pollutants [20]. The high drug‐loading capacity and pH‐responsive release properties make ZIF‐8 a promising candidate for drug delivery (e.g., doxorubicin). Additionally, ZIF‐8 can adsorb heavy metals (e.g., Pb^2+^ and Cd^2+^) and organic pollutants (e.g., dyes and pesticides) for wastewater treatment. Therefore, as a novel drug delivery system, ZIF‐8 is highly compatible with the weakly acidic microenvironment commonly found in diabetic wounds, providing an ideal platform for constructing an “intelligent” targeted drug release system [21]. Although ZIF‐8 has been extensively studied for the delivery of anticancer drugs, its application in delivering SalB, a naturally active compound, for the treatment of diabetic wounds remains a cutting‐edge and underexplored area. Previous therapeutics focus solely on monotherapy, the synthesis of ZIF‐8 or the pharmacological effects of SalB. Therefore, studies developing a system with synergistic functions to validate their synergistic effects in promoting angiogenesis and modulating inflammation in the complex diabetic wound microenvironment are lacking.

**FIGURE 1:**
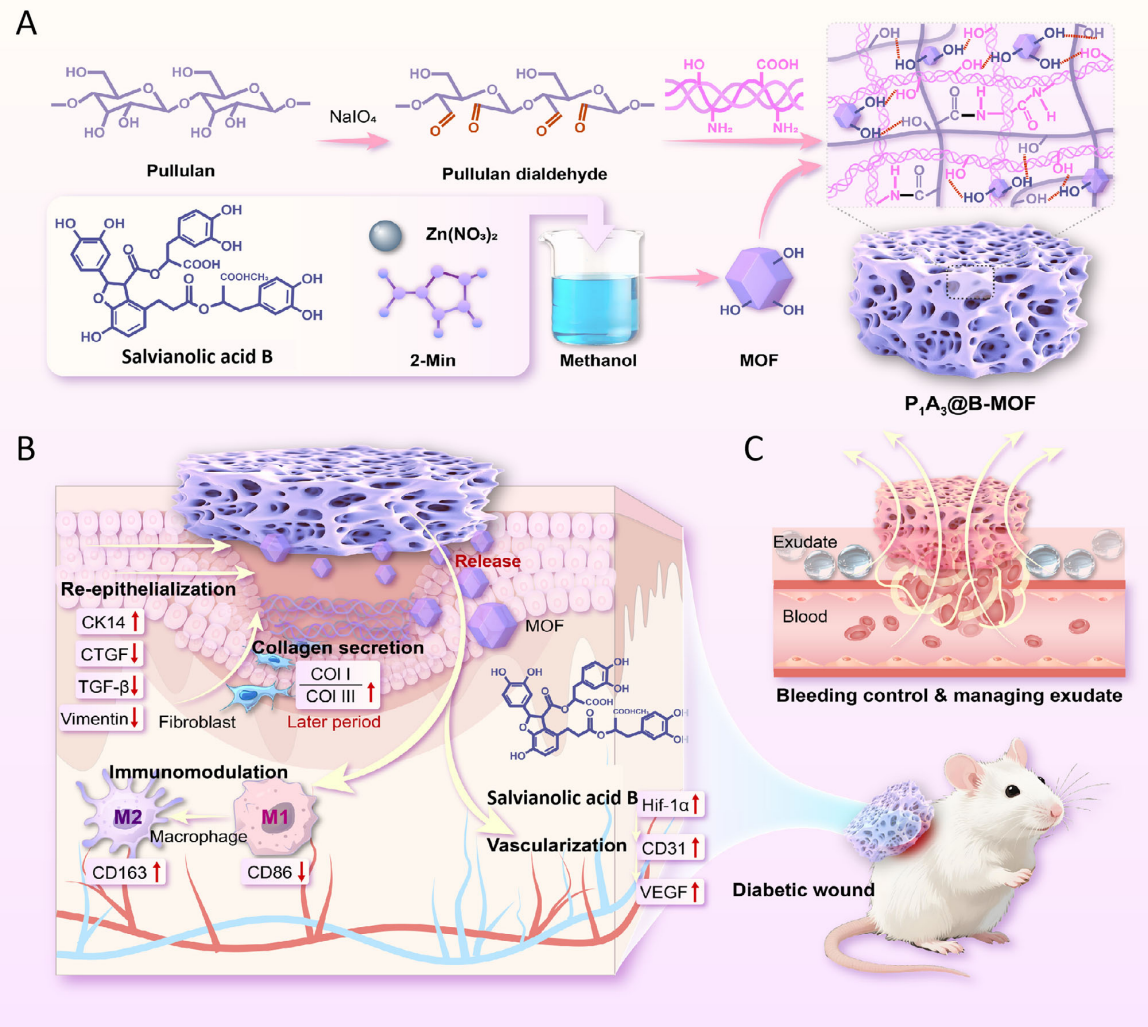
Preparation and application of oxidized polysaccharide‐decellular dermal matrix sponge with in situ assembled B‐MOF. (A) Preparation of the P_1_A_3_@B‐MOF sponge. (B) Regulation of the diabetic wound healing microenvironment by the P_1_A_3_@B‐MOF sponge. (C) Hemostasis and wound fluid management using the P_1_A_3_@B‐MOF sponge.

Pullulan (Pu) is a natural, water‐soluble microbial exopolysaccharide produced by the fermentation of *Aureobasidium pullulans*. It consists of repeating maltotriose units linked by α‐(1→6)‐glycosidic bonds. This unique structure endows Pu with excellent film‐forming, adhesive, and plasticizing properties [22]. Moreover, Pu dissolves rapidly in both cold and hot water, forming solutions with low viscosity and good fluidity. Consequently, Pu has rarely been reported as a sponge dressing for wound treatment, being primarily employed in candy coatings, facial masks, and hydrogel products to provide a smooth texture and act as a carrier for active ingredients [23]. In light of the above, this study successfully developed and validated a polysaccharide‐based shape‐memory sponge dressing integrated with B‐MOF (P_1_A_3_@B‐MOF), offering an innovative spatiotemporally controlled combination strategy for the management of diabetic wounds. First, the hydroxyl groups of water‐soluble Pu were oxidized by sodium periodate to form aldehyde groups, which then reacted with the amino groups of collagen in the porcine acellular dermal matrix(PADM) via a Schiff base reaction, resulting in an intermolecular interpenetrating network (P_1_A_3_). Subsequently, SalB‐loaded MOF was generated in situ within the P_1_A_3_ sponge through self‐assembly, and the composite was freeze‐dried to form an exudate‐triggered shape‐memory sponge for the comprehensive management of diabetic wounds (Figure 1). The physicochemical properties, pore structure, degradation behavior, and drug release profile of the P_1_A_3_@B‐MOF sponge were analyzed, confirming its excellent active exudate management capacity. In vitro cell experiments further demonstrated that the sponge extract modulated macrophage polarization, attenuated inflammatory responses, and promoted the migration and angiogenic activity of human umbilical vein endothelial cells (HUVECs). Moreover, in a diabetic rat model, P_1_A_3_@B‐MOF alleviated wound inflammation, stimulated granulation tissue formation and vascular reconstruction, enhanced epithelialization and collagen deposition, reduced scar formation, and accelerated the wound healing rate. Therefore, this multifunctional sponge, which combines immunomodulatory and proangiogenic properties, demonstrates great potential for promoting skin wound healing and offers a novel therapeutic avenue for diabetic wounds.

## 2 Results and Discussion

### 2.1 Preparation of Oxidized Pullulan‐Acellular Dermal Matrix (PA) Sponge

Pu, a linear polysaccharide derived from the fungus *Aureobasidium pullulans*, consists of maltotriose units linked by α‐1,6‐glycosidic bonds [24]. It has abundant modifiable pendant hydroxyl groups that can be oxidized to form aldehyde groups. The biocompatibility, degradability, and renewability of Pu make it ideal for use as a wound dressing agent. Its viscosity, film‐forming ability, and oxygen barrier properties also render it suitable as an adhesive in the food, pharmaceutical, and agricultural industries [25]. However, its high water solubility limits its applications.

**FIGURE 2:**
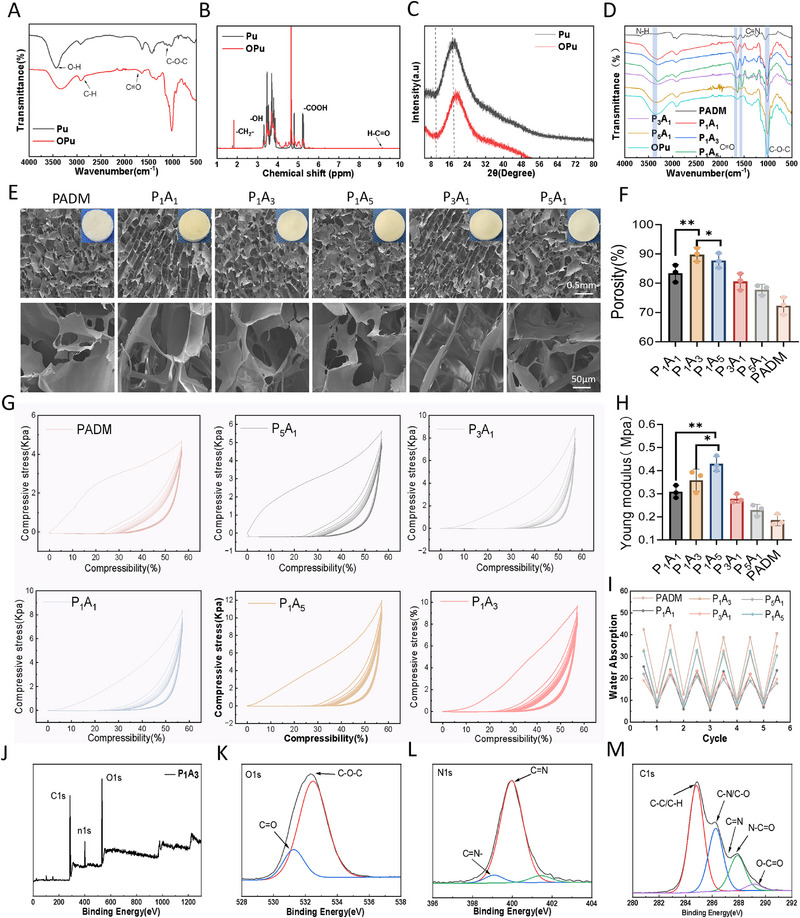
Preparation and characterization of The PA sponge. (A) FTIR spectrum. (B) ^1^H NMR spectrum. (C) X‐ray diffraction (XRD) pattern. (D) FTIR spectrum. (E) Microstructure of the sponge. (F) Porosity. (G) Compressive stress–strain curve. (H) Compressive modulus. (I) Cyclic water absorption curve. (J) XPS spectrum. (K) O1s spectrum. (L) N1s spectrum. (M) C1s spectrum. Data (mean ± SD) were obtained from three independent experiments and analyzed using one‐way ANOVA with Tukey's post hoc multiple comparison test (*p < 0.05, **p < 0.01, ***p < 0.001).

In this study, Pu was oxidized with NaIO_4_ at room temperature in the dark to yield OPu. Subsequently, this product was cross‐linked with a collagen‐rich acellular dermal matrix via a Schiff base reaction, forming an interpenetrating network. SalB was loaded into the MOF and incorporated into this network through hydrogen bonds and ionic interactions, followed by freeze‐drying to fabricate the sponge (Figure 1). As illustrated in Figure 2A, both Pu and OPu samples exhibited infrared spectral bands characteristic of maltotriose, including hydroxyl group stretching at 3400 cm^−1^, methylene (─CH_2_─) stretching at 2930 cm^−1^, C─O stretching at 1018 cm^−1^, α‐1,4‐glycosidic linkage at 1154 cm^−1^, and α‐1,6‐glycosidic linkage at 928 cm^−1^ [26]. Although the Fourier‐transform infrared (FTIR) spectra of Pu and OPu are similar, a new absorption peak appears in the OPu sponge at 1727 cm^−1^, attributed to the carbonyl stretching vibration of aldehyde groups. The ^1^H NMR spectra (Figure 2B) show resonances for hemiacetal protons between 4.5 and 5.6 ppm and a distinct aldehyde proton signal between 9.0 and 9.5 ppm in the OPu. Cleavage of the C2─C3 bond correlated with the reduced intensity of the H3 (approximately 3.8 ppm) and H4 (approximately 3.5 ppm) signals, while the H2 protons adjacent to the aldehyde shifted downfield to 4.5–5.5 ppm. The X‐ray diffraction patterns (Figure 2C) indicate a reduction in the original crystalline peak at 2*θ* = 13° and an increase in the amorphous halo near 2*θ* = 20°, consistent with the disrupted glucose unit arrangement post‐C2–C3 cleavage [27]. The formation of oxidized aldehyde and carboxyl groups introduces structural disorder, hindering chain packing and resulting in a predominantly amorphous material with enhanced chain mobility [28].

Fourier‐transform infrared spectroscopy identified characteristic signals of ADM, including amide I (1630–1660 cm^−1^), amide II (1530–1550 cm^−1^), and hydroxyl groups (3300–3400 cm^−1^) (Figure 2D). OPu exhibited aldehyde peaks at 1720–1740 cm^−1^, carboxyl groups between 1600 and 1650 cm^−1^, and carbohydrate‐related bands in the 1000–1150 cm^−1^ range [29]. Notably, the composite scaffold spectrum displayed a distinctive imine (Schiff base) peak at 1640–1680 cm^−1^, alongside increased intensities of amide I (1650 cm^−1^) and amide II (1540 cm^−1^) bands. These spectral changes, coupled with the corresponding decrease in the aldehyde peak at 1720 cm^−1^, serve as evidence for cross‐linking mediated by Schiff base and amide bond formation [30].

As shown in Figure 2E, the PA sponge appears milky white and exhibits an interconnected, fluffy porous structure, with intact pore architecture and porosity greater than 75%. Among the groups, the P_1_A_3_ sponge shows the highest porosity at 87 ± 2.5% (Figure 2F). As depicted in Figure 2I, with the increasing content of OPu, the water absorption rate of the sponge gradually decreases. The P_1_A_3_ group demonstrated excellent water absorption performance compared to the other groups. The water absorption rate can reach 38 ± 1.4 times its own mass. Moreover, even after five cycles of water absorption, the sponge maintains a stable water absorption rate. This characteristic enables the sponge to rapidly absorb exudate from wounds and effectively promote the drainage of exudate from the wound, storing it within the scaffold pores to create a microwet environment conducive to healing and favorable for in vivo applications [27, 31].

As shown in Figure S1A, all sponge groups exhibit hydrophilic characteristics, with water contact angles decreasing over time. However, as the content of OPu increases, the water contact angle gradually increases (Figure S1B). This change may be attributed to the Schiff base reaction, which consumes the hydrophilic groups (─NH_2_) from collagen and the aldehyde groups from OPu (which can further hydrate into hydrophilic glycol), leading to an overall decrease in hydrophilicity [32].

The compression mechanical results of the sponges are shown in Figure 2G. After 15 successive compressions, all sponge groups display good recovery performance. The maximum stress during the first compression cycle of PADM reaches 5.21 kPa, with a compressive modulus of 0.18 ± 0.1 MPa. With the addition of oxidized pullulan, the maximum stress of the sponge gradually increases, with the P_1_A_3_ sponge achieving the highest maximum stress of 10.8 kPa and a compressive modulus of 0.41 ± 0.12 MPa (Figure 2H). Furthermore, after multiple cycles, the stress–strain curves overlap significantly, with no evident hysteresis loops, indicative of excellent shape memory stability and fatigue resistance. This improved performance may be due to the formation of aldehyde groups (─CHO) during the oxidation of OPu, which can react with the free amino groups (─NH_2_) of collagen in PADM through a Schiff base reaction, resulting in a covalent cross‐linked network. As the ratio of OPu increases, the cross‐linking density enhances, leading to increased intermolecular interactions and, consequently, improved mechanical strength [33].

Figure 2J shows the XPS survey spectrum of the P_3_A_1_ sponge. In the O 1s spectrum (Figure 2K), the shift or intensity change of the carboxyl peak reflects electrostatic interactions or salt‐bridge formation, providing direct evidence for chemical cross‐linking, such as Schiff base formation, between oxidized pullulan and the acellular dermal matrix. In the N 1s spectrum (Figure 2L), the free ─NH_2_ peak is weakened, and a peak possibly attributable to the imine bond appears near 398.5 eV. In the C1s spectrum (Figure 2M), a clear reduction in the aldehyde/carboxyl peaks is observed, accompanied by the appearance of new peaks corresponding to imine bonds (─C═N─) or ester bonds (O─C═O). In summary, compared with the other formulations, P_1_A_3_ exhibits relatively superior physicochemical properties.

### 2.2 Preparation and Characterization of P_1_A_3_@B‐MOF Sponge

**FIGURE 3:**
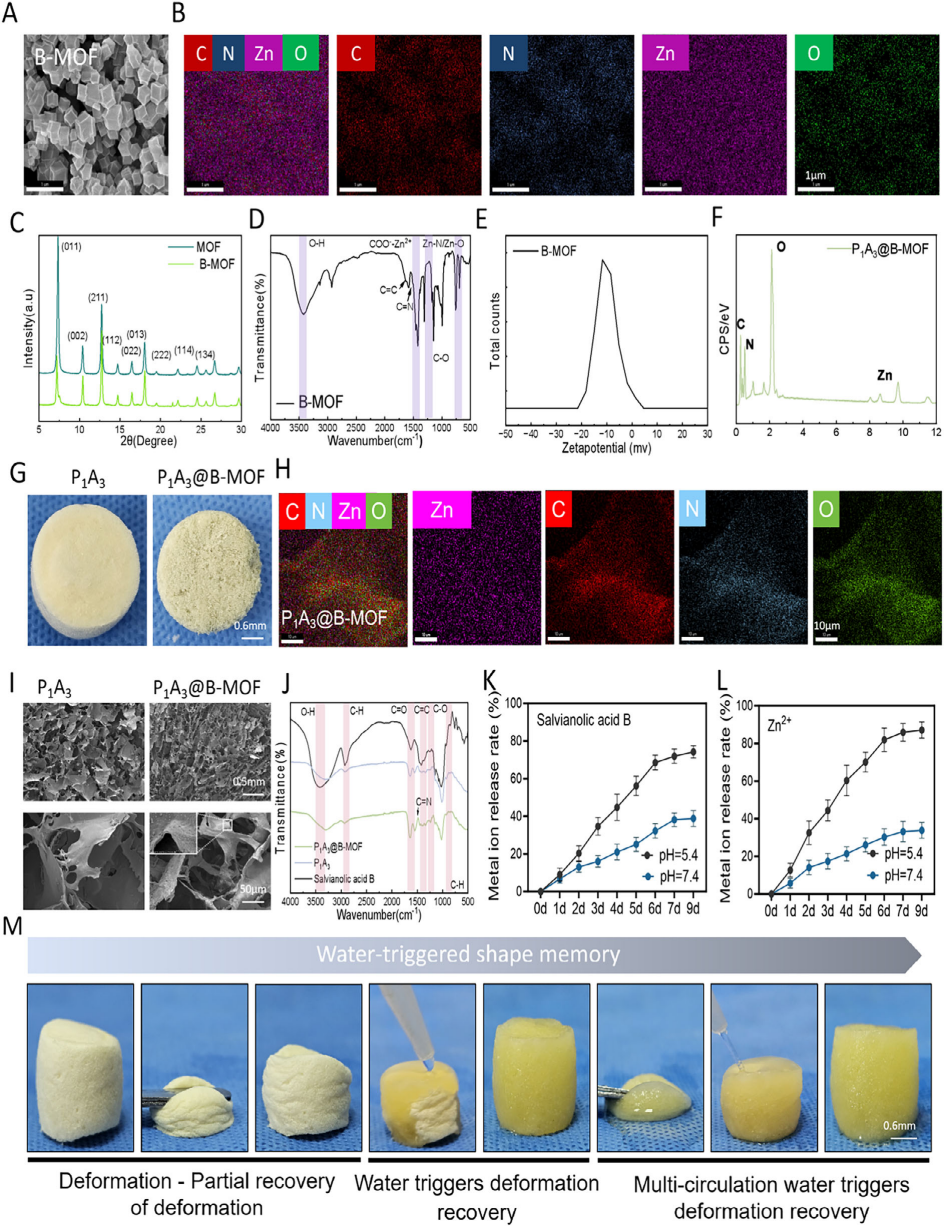
Preparation and Characterization of P_1_A_3_@B‐MOF Sponge. (A) SEM image of B‐MOF. (B) Elemental mapping image. (C) XRD analysis. (D) Fourier‐transform infrared spectroscopy of B‐MOF. (E) Zeta potential measurements. (F) EDS spectrum. (G) Morphological images of the sponge. (H) Elemental mapping image of P_1_A_3_@B‐MOF. (I) SEM image of P_1_A_3_@B‐MOF. (J) Fourier‐transform infrared spectroscopy (FTIR) of P_1_A_3_@B‐MOF. (K) Time‐release curve of SalB. (L) Time‐release curve of Zn^2+^. (M) Water‐triggered shape memory performance of the P_1_A_3_@B‐MOF sponge.

The integration of SalB into MOFs, particularly ZIF‐8, enhances stability and enables sustained release, thereby mitigating rapid degradation [27]. ZIF‐8's biocompatibility and pH‐sensitive release facilitate targeted delivery to slightly acidic microenvironments, amplifying provascularization effects [34]. Figure 3A presents a SEM image revealing the rhombic dodecahedral morphology, smooth surfaces, and uniform particle size distribution (100–200 nm) of B‐MOF, consistent with ZIF‐8's crystallinity [16, 18]. Elemental analysis via Energy Dispersive X‐ray Spectrometer (EDS) (Figure 3B) confirmed that the B‐MOF comprised C, Zn, N, and O.

The X‐ray diffraction (XRD) pattern of pure ZIF‐8 (Figure 3C) shows distinct peaks at 2*θ* = 7.3°, 10.4°, 12.7°, 14.7°, 16.4°, 18°, 22.1°, 24.5°, and 26.7°, corresponding to the (011), (002), (112), (022), (013), (222), (114), (233), and (134) planes, respectively [19]. These results are consistent with the simulated ZIF‐8 structure, confirming its high crystallinity and characteristic sodalite topology. Loading SalB into the ZIF‐8 framework minimally affected its structural integrity. The positions of the primary diffraction peaks, such as those at 7.3° and 10.4°, remained largely unchanged, indicating the stability of the ZIF‐8 crystallinity [17, 20]. However, a slight reduction in the intensity of certain peaks, including those at 12.7° and 16.4°, was observed, which can be attributed to the incorporation of SalB molecules into the ZIF‐8 pores, which may have altered the diffraction intensities of the specific crystal planes. XRD analysis confirmed that the SalB@ZIF‐8 composite maintained the crystalline integrity of the ZIF‐8 framework, with only minor lattice perturbations caused by the presence of SalB molecules. Importantly, no phase transition or structural collapse was induced during the drug loading process. This finding underscores the stability of ZIF‐8 as a potential drug carrier and provides a structural basis for the observed sustained and pH‐responsive release behavior of SalB, a feature advantageous for wound healing applications.

The Fourier‐transform infrared spectrum of B‐MOF (Figure 3D) reveals its key interactions with the substrate. The ─OH peak shifted from 3400–3200 to 3350 cm^−1^, indicating hydrogen bonding or coordination between the ─OH groups and ZIF‐8's imidazole nitrogen or Zn^2+^. The C═O peak shifted from 1700 to 1650–1600 cm^−1^, suggesting coordination bonding between SalB's carboxyl groups and ZIF‐8's Zn^2+^, forming zinc carboxylate salts. The C═N peak shifted from 1585 to 1575 cm^−1^, likely due to hydrogen bonding between SalB's ─OH groups and the imidazole nitrogen [16, 18]. As depicted in Figure 3E, the zeta potential of B‐MOF was approximately ─13 mV, in contrast to the typical positive potential of +10 to +30 mV for ZIF‐8, which is dominated by zinc ions. This observed shift is likely attributed to the incorporation of SalB into the porous ZIF‐8 framework, which shields the internal positive charges and results in weak coordination between the oxygen atoms of SalB and the Zn^2+^ centers, along with the presence of deprotonated carboxylic acid groups (─COO^−^) that contribute to the negative charge [12]. Visual observation of the samples (Figure 3G) showed that the P_1_A_3_ sponge was off‐white, whereas the P_1_A_3_@B‐MOF sponge exhibited a pale yellow color after the in situ assembly of the B‐MOF component. Elemental mapping analysis (Figure 3F,H) confirmed the successful incorporation and uniform distribution of B‐MOF within the sponge matrix, which was primarily composed of C, Zn, N, and O elements. The internal structure of the P_1_A_3_@B‐MOF sponge presented a loose, porous network with B‐MOF uniformly distributed on the surface of the pore walls (Figure 3I).

The FTIR spectrum (Figure 3J) shows the overlapping O─H/N─H peak at 3300 cm^−1^, the C═O peak of OPu at 1720 cm^−1^, and the amide I/II peaks of PADM at 1650/1540 cm^−1^. Metal ion release experiments (Figure 3K,L) indicated that both SalB and Zn^2+^ were released consistently at pH = 7.4 and 5.4, with a faster release in acidic conditions (pH = 5.4), exceeding 70% within 7 days. The pH‐responsive release of P_1_A_3_@B‐MOF can be attributed to its unique coordination chemistry and carrier properties. Under acidic conditions (pH = 5.4), protonation of the imidazole nitrogen atoms (N → NH^+^) in the 2‐methylimidazole ligands occurs, which weakens their coordination with Zn^2+^ [35]. Concurrently, the deprotonated carboxylic acid groups (─COO─) of SalB become protonated (─COOH), diminishing their electrostatic interactions with the metal framework. This resulted in framework disintegration and SalB release.

For the shape memory effect of the sponge, water serves as the triggering switch. The introduction of water lowers the activation energy of the sponge's dynamic bonds, allowing for the release of the prestored elastic strain energy within the material [36]. This drives the sponge to purposefully and actively recover to a predefined permanent shape, following an energy‐driven process characterized by predetermined pathways and endpoints [37]. As shown in Figure 3M, the P_1_A_3_@B‐MOF sponge undergoes a shape‐fixation programming process, typically involving dehydration and cooling, which locks in the temporary shape. Upon triggering, the sponge material accurately returns to its original permanent shape as manufactured, rather than any arbitrary shape [38]. The deformation‐fixation‐recovery cycle enables multiple shape cycling, with each recovery leading to the same endpoint [39]. This confirms that the sponge scaffold possesses excellent shape memory functionality, allowing it to revert to its compressed state upon liquid‐triggered activation [40]. Thus, it can conform to irregular wounds, providing patients with a comfortable experience.

To evaluate the stability of the P_1_A_3_ and P_1_A_3_@B‐MOF sponges under enzymatic and acidic conditions, we assessed their degradation in collagenase solution and a buffer solution at pH = 5.4. As illustrated in Figure S2A, both P_1_A_3_ and P_1_A_3_@B‐MOF sponges exhibited rapid and sustained degradation characteristics in the collagenase solution, with nearly complete degradation achieved within 96 h. However, the degradation rate of P_1_A_3_@B‐MOF was significantly slower compared to that of P_1_A_3_. Specifically, after 96 h, the degradation rate of the P_1_A_3_ sponge approached 90%, while the P_1_A_3_@B‐MOF sponge exhibited a more gradual degradation profile, reaching approximately 75% degradation by this time point. In the buffer solution at pH = 5.4, the degradation rate of P_1_A_3_@B‐MOF was also significantly slower compared to that of the P_1_A_3_ sponge (Figure S2B). These results clearly indicate that the incorporation of B‐MOF substantially slows down the degradation of the sponge, likely due to the enhanced structural stability imparted by the B‐MOF. As a metal–organic framework material, B‐MOF possesses inherent mechanical strength and chemical stability. When combined with the sponge matrix, it may improve the overall material density through interfacial interactions or cross‐linking effects, thereby delaying the penetration and diffusion of degradation media such as water or enzymes [41]. Additionally, the metal nodes in B‐MOF may form coordination interactions with the polymer chains of the sponge, further inhibiting the hydrolysis or enzymatic degradation processes, which leads to a more gradual degradation behavior. In contrast, the unmodified P_1_A_3_ sponge has a relatively loose structure that allows degradation media to penetrate and disrupt its internal architecture more easily, resulting in a faster degradation kinetics.

### 2.3 Biocompatibility Evaluation

**FIGURE 4:**
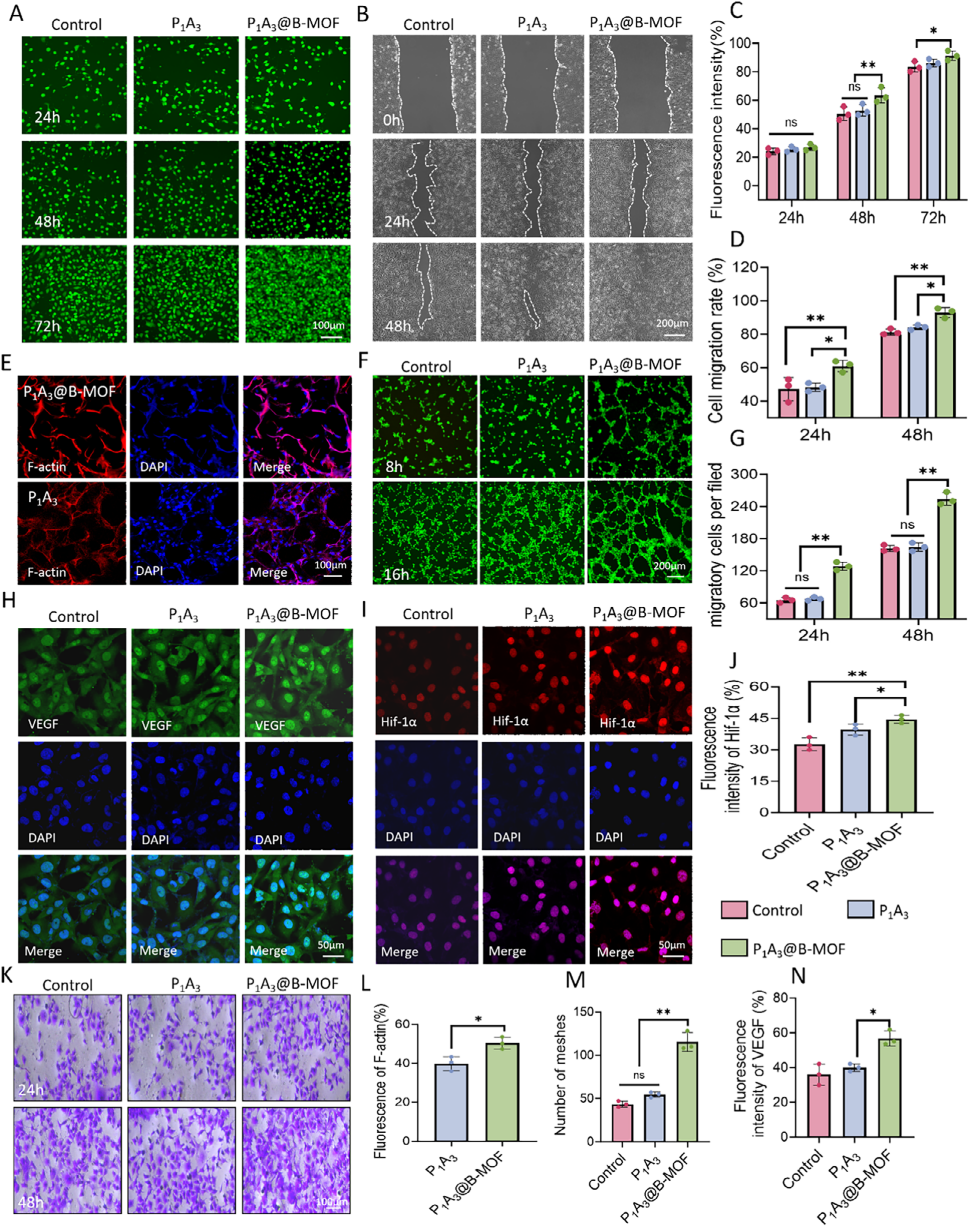
Evaluation of the biocompatibility and proangiogenic capacity of P_1_A_3_@B‐MOF sponge. (A) Representative images of live/dead cell fluorescence staining. (B) Images of the cell migration assay. (C) Quantitative analysis of F‐actin immunofluorescence images. (D) Cell migration rates. (E) Fluorescence images of the F‐actin‐stained cells. (F) Images of the tube formation assay. (G) Number of invading cells. (H) Immunofluorescence staining image showing VEGF expression. (I) Immunofluorescence staining images of Hif‐1α. (J) Quantitative analysis of Hif‐1α immunofluorescence. (K) Images of the HUVEC invasion assay. (L) Quantitative analysis of F‐actin immunofluorescence. (M) Quantitative analysis of mesh number. (N) Quantitative analysis of VEGF immunofluorescence. Data (mean ± SD) were obtained from three independent experiments and analyzed using one‐way ANOVA with Tukey's post hoc multiple comparison test (*p < 0.05, **p < 0.01, ***p < 0.001).

Biocompatibility is crucial for biomaterials used in clinical practice. We evaluated sponge cytotoxicity using live/dead cell dual staining with different concentrations of the sponge extract. Figure 4A shows that all groups displayed numerous bright green fluorescent signals with notably elongated spindle‐like structures, indicating excellent cytocompatibility. Notably, the P_1_A_3_@B‐MOF group demonstrated a significantly high cell proliferation rate. Quantitative fluorescence intensity analysis (Figure 4C) confirmed a statistically significant difference (*p* < 0.01). The cell scratch assay, a prevalent method for assessing cell migration and repair, mimics in vivo wound closure. After 48 h, the migration rates of HUVECs were 78.5 ± 2.8% for the control, 83.4 ± 2.5% for P_1_A_3_, and 92.7 ± 2.2% for the P_1_A_3_@B‐MOF group (Figure 4B,D), highlighting that P_1_A_3_@B‐MOF substantially enhances HUVEC proliferation and migration. Adhesion assays demonstrated robust HUVECs attachment to both P_1_A_3_ and P_1_A_3_@B‐MOF sponges, with the cells predominantly displaying a spindle shape (Figure 4E), indicating the effectiveness of the fibrous scaffold in supporting cell growth. Quantitative fluorescence analysis (Figure 4L) confirmed significantly greater cell adhesion to the P_1_A_3_@B‐MOF sponge (*p* < 0.05).

Angiogenesis refers to the formation of new blood vessels from preexisting vasculature and involves endothelial cell migration, proliferation, and lumen formation. The tube formation assay mimics capillary generation in vivo. Compared to the control group, P_1_A_3_@B‐MOF exhibited angiogenic potential as early as 8 h (Figure 4F) and formed a denser vascular network by 16 h (Figure 4M). To investigate this mechanism, we assessed the expression of VEGF, a key regulator of angiogenesis. Figure 4H shows low VEGF expression in HUVECs in both Control and P_1_A_3_ groups under in vitro conditions. However, coculture with the P_1_A_3_@B‐MOF group significantly increased VEGF fluorescence (Figure 4N) (*p* < 0.01), highlighting the P_1_A_3_@B‐MOF sponge's proangiogenic potential. Furthermore, Hif‐1α is crucial for angiogenesis, especially under hypoxic conditions, by activating proangiogenic factors [42]. As depicted in Figure 4I, the P_1_A_3_@B‐MOF group exhibited the strongest Hif‐1α fluorescence signal (43.4 ± 2.8%) (Figure 4J), consistent with the VEGF staining results. Collectively, these findings indicate that the P_1_A_3_@B‐MOF sponge is highly cytocompatible and that SalB release effectively enhances angiogenesis and VEGF secretion. The invasion assay mimics the behavior of endothelial cells crossing the basement membrane or extracellular matrix, directly reflecting key steps in angiogenesis, specifically the migration and invasion capabilities of endothelial cells [43]. As shown in Figure 4K, using the crystal violet staining migration assay, compared with the control group, HUVECs in the P_1_A_3_@B‐MOF group demonstrated invasive potential as early as 24 h and formed a denser layer of invading HUVECs by 48 h (Figure 4G).

### 2.4 Evaluation of Immunomodulatory Ability of P_1_A_3_@B‐MOF Sponge

**FIGURE 5:**
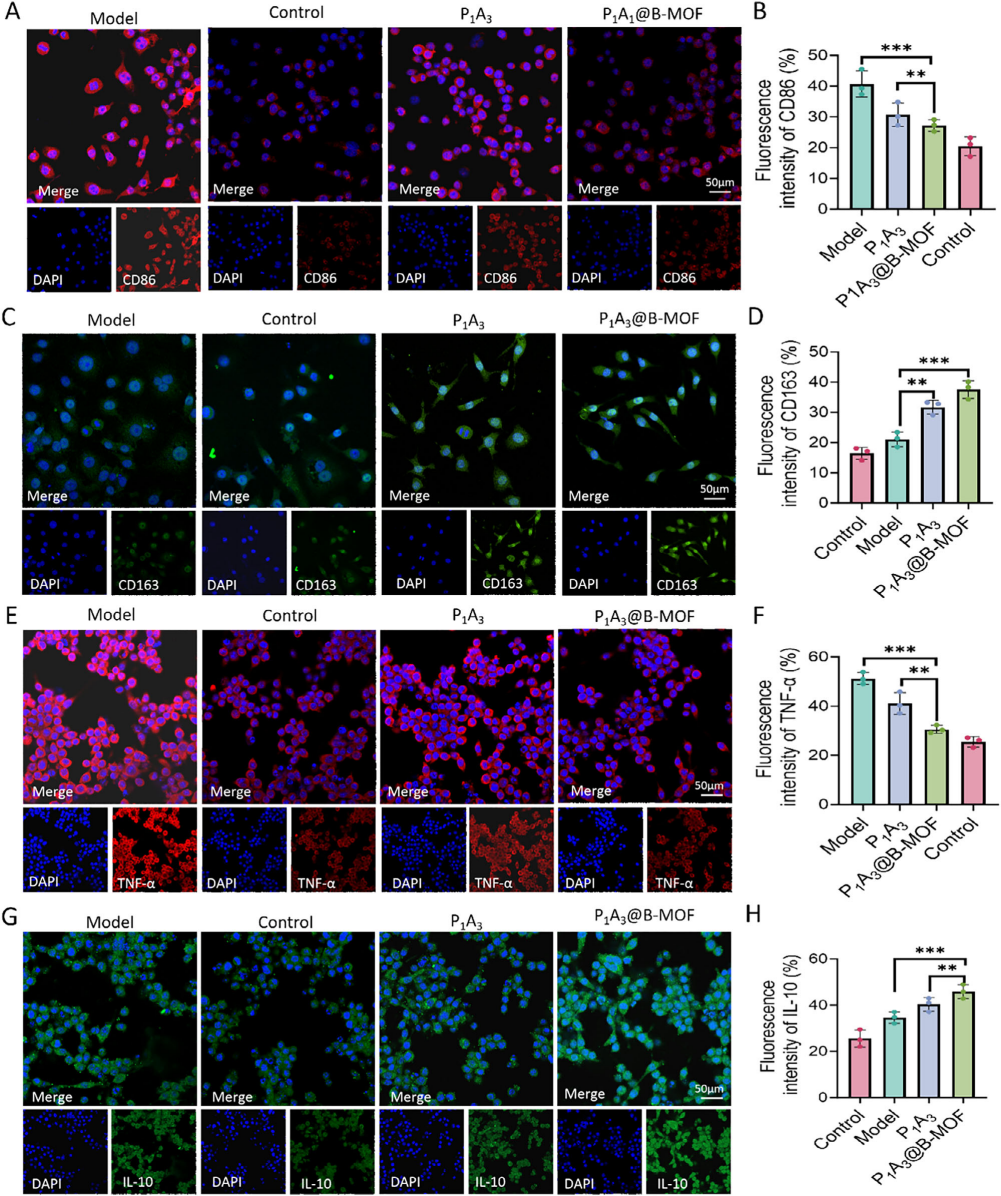
Evaluation of the immunomodulatory and anti‐inflammatory capacities of the P_1_A_3_@B‐MOF sponge. (A) Immunofluorescence staining for CD86. (B) Quantitative analysis of CD86 immunofluorescence. (C) Immunofluorescence staining for CD163. (D) Quantitative analysis of CD163 immunofluorescence signal. (E) Immunofluorescence staining for TNF‐α. (F) Quantitative analysis of TNF‐α immunofluoress. (G) Immunofluorescence staining for IL‐10. (H) Quantitative analysis of IL‐10 immunofluorescence. Data (mean ± SD) were quantified from three independent experiments and analyzed using one‐way ANOVA with Tukey's post hoc multiple comparison test (*p < 0.05,** *p* < 0.01, ***p < 0.001).

The anti‐inflammatory properties of the P_1_A_3_@B‐MOF sponge were evaluated using a macrophage inflammation model. The primary objective was to examine the influence of P_1_A_3_ and P_1_A_3_@B‐MOF sponge extracts on macrophage polarization through coculture with RAW264.7 macrophages. As shown in Figure 5A, the P_1_A_3_@B‐MOF group exhibited a significant reduction in the expression of the M1 macrophage marker CD86 compared to the P_1_A_3_ and Control groups (Figure 5B). These findings demonstrate that P_1_A_3_@B‐MOF effectively promoted the polarization of macrophages from a proinflammatory M1 state to an anti‐inflammatory M2 phenotype. Specifically, a significant increase in the expression of the M2 marker CD163 was observed (*p* < 0.01) (Figure 5C,D), indicating a shift toward the M2 macrophage state. In contrast, unstimulated macrophages in the control group exhibited a weak TNF‐α expression profile, characteristic of the M0 phenotype (Figure 5E). However, the LPS‐induced model group showed marked changes in RAW264.7 macrophage morphology and a peak in TNF‐α fluorescence expression, indicative of M1 macrophage polarization (Figure 5F). Notably, when these M1‐polarized macrophages were cocultured with the P_1_A_3_@B‐MOF extract, TNF‐α fluorescence expression significantly decreased, whereas IL‐10 fluorescence expression significantly increased (Figure 5G,H). These results demonstrate that P_1_A_3_@B‐MOF promotes the polarization of macrophages toward the anti‐inflammatory M2 phenotype, which is expected to alleviate inflammation and create a more favorable environment for wound healing.

The immunomodulatory properties of the P_1_A_3_@B‐MOF sponge likely arise from the synergistic effects of its two principal components: as a zinc‐based MOF, the degradation of B‐MOF releases Zn^2+^, which can inhibit the NF‐κB and NLRP3 inflammasome pathways, thereby downregulating the expression of proinflammatory factors [44]. Concurrently, Zn^2+^ can activate the STAT3/STAT6 signaling axis, facilitating the polarization of M1 macrophages toward an M2 phenotype [45]. Additionally, the incorporated SalB compound exerts immunomodulatory effects by inhibiting the NF‐κB and MAPK pathways, leading to a reduced expression of M1‐associated factors, such as CD86 [46]. SalB activates PPARγ and STAT3, promoting the expression of M2 markers, such as CD163 [47].

### 2.5 Evaluation of Antibacterial Performance of P_1_A_3_@B‐MOF Sponge

Hyperglycemic conditions create a favorable environment for microbial proliferation, and the peripheral neuropathy and vascular complications commonly associated with diabetes further impair the chemotaxis and phagocytic functions of local immune cells, making it difficult to effectively eliminate pathogens [48]. This susceptibility renders diabetic wounds prone to persistent bacterial infections. Therefore, applying dressings with broad‐spectrum antibacterial properties is strictly critical [49]. Such dressings can actively disrupt this vicious cycle by effectively inhibiting or eliminating various Gram‐positive and Gram‐negative bacteria, directly controlling local wound infections and reducing inflammatory responses, thereby providing essential support for the transition of wounds from the inflammatory phase to the proliferative phase.

In this study, *Escherichia coli* (*E. coli*) and *Staphylococcus aureus* (*S. aureus*) were selected as representative microbial strains to systematically evaluate the in vitro antibacterial performance of P_1_A_3_@B‐MOF. The antibacterial effects were quantified using the agar plate count method, with morphological observations also conducted. As shown in Figure S3A, the P_1_A_3_ group contained a substantial number of surviving *S. aureus* and *E. coli*, with inhibition rates of (5.8 ± 3.1)% and (6.4 ± 2.7)%, respectively. In contrast, the P_1_A_3_@B‐MOF group showed almost no survival of *S. aureus* and *E. coli*, with significantly increased inhibition rates of (90.4 ± 4.3)% and (92.2 ± 3.6)% (Figure S3C,D). To visualize the bactericidal effect of P_1_A_3_@B‐MOF more intuitively, live/dead staining kits were used to assess the cocultured bacteria. As illustrated in Figure S3B, the control group and the P_1_A_3_ group exhibited extensive green fluorescence (indicating viable bacteria). Conversely, the P_1_A_3_@B‐MOF group displayed significant areas of red fluorescence, indicating a marked increase in the number of dead *S. aureus* (Figure S3E) and *E. coli* (Figure S3F), with quantitative fluorescence statistics further supporting these findings (*p* < 0.05).

The superior antibacterial efficacy of P_1_A_3_@B‐MOF is attributed to synergistic mechanisms, primarily involving biochemical killing mediated by metal ions and physical inhibition stemming from its material structure. At the ionic level, released Zn^2+^ disrupt the integrity of bacterial cell membranes by binding to phospholipids and thiol groups (─SH), leading to membrane potential collapse and content leakage [50]. Additionally, Zn^2+^ interferes with bacterial energy metabolism by competitively binding to key metabolic enzymes, such as dehydrogenases. On the structural level, the 3D porous network of the P_1_A_3_@B‐MOF sponge physically captures and confines bacteria, effectively limiting their migration and diffusion [51]. The combined action of these chemical bactericidal mechanisms and physical blocking strategies endows the material with efficient and broad‐spectrum antibacterial activity.

### 2.6 Evaluation of In Vitro Hemostatic Performance of P_1_A_3_@B‐MOF Sponge

**FIGURE 6:**
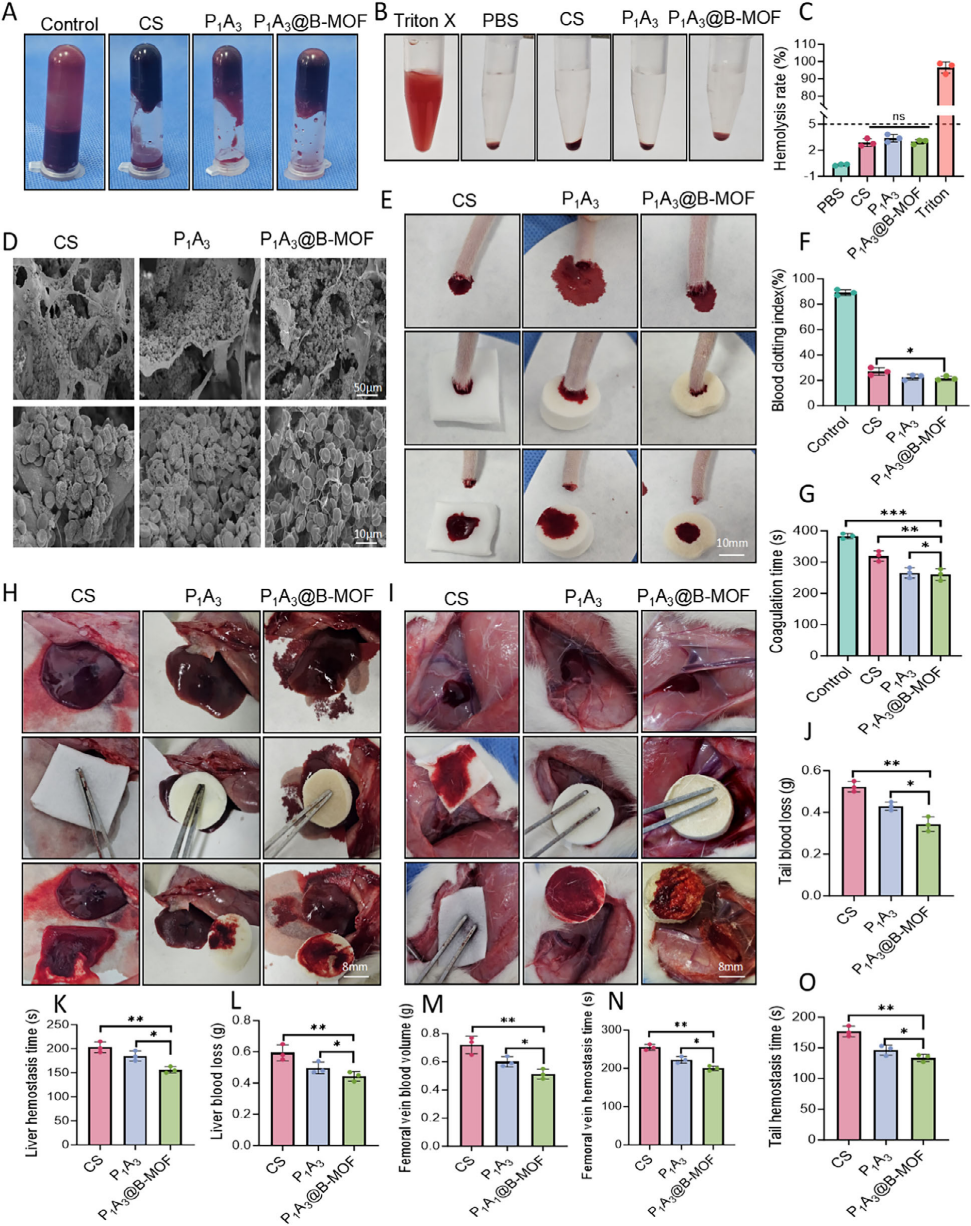
Evaluation of the in vitro and in vivo hemostatic performance of the P_1_A_3_@B‐MOF sponge. (A) Images of in vitro blood clotting. (B) Hemolysis assay image. (C) Hemolysis rate. (D) Microscopic images of the sponge after incubation with anticoagulated whole blood. (E) Images of rat tail bleeding model. (F) Coagulation index. (G) Coagulation time. (H) Images of rat liver hemorrhage model. (I) Images of rat femoral vein hemorrhage model. (J) Total blood loss in the tail‐amputation model. (K) Hemostasis time in the liver‐injury model. (L) Total blood loss in the liver injury model. (M) Total blood loss in the femoral vein injury model. (N) Hemostatic time in the femoral vein injury model. (O) Hemostatic time in the tail amputation model. Data (mean ± SD) were obtained from three independent experiments and analyzed using one‐way ANOVA with Tukey's post hoc multiple comparison test (*p < 0.05, **p < 0.01, ***p < 0.001).

In vitro clotting time assays demonstrated the hemostatic function of the sponge (Figure 6A). Sponges from the P_1_A_3_ and P_1_A_3_@B‐MOF groups formed firm blood clots, whereas the CS group exhibited partial coagulation failure. Notably, the P_1_A_3_@B‐MOF group exhibited the shortest clotting time, which was significantly shorter than that of the CS group (Figure 6G). Hemolysis assessment using rat‐derived blood cells revealed lower rates across all sponge groups compared to controls, with the P_1_A_3_ and P_1_A_3_@B‐MOF groups both below 5% (Figure 6B,C). Furthermore, the blood coagulation index (BCI) results (Figure 6F) showed lower values across all groups, with the P_1_A_3_@B‐MOF group recording the lowest value of 16.7 ± 2.1%, indicating a superior hemostatic performance. Blood cell adhesion morphology (Figure 6D) demonstrated that the larger and more porous structure of the P_1_A_3_@B‐MOF sponges facilitated the observation of numerous blood cell aggregates within the sponge framework. The intricate porous structure of hemostatic sponges interacts with blood platelets, accelerating thrombus formation to effectively seal wounds and achieve rapid haemostasis [31, 52]. Clinically, these hemostatic sponges are widely used for rapid bleeding control, inhibition of bacterial growth to prevent infection, and promotion of wound healing [53].

The rat tail bleeding model was used to evaluate the hemostatic effect of the sponges. All sponge groups achieved hemostasis (Figure 6E). The P_1_A_3_@B‐MOF sponge group showed significantly reduced blood loss (0.34 ± 0.04 g) (Figure 6J) and shorter bleeding time (142 ± 5 s) than the CS group, with these differences being statistically significant (*p* < 0.05) (Figure 6O). In a rat liver injury model, the hemostatic performances of a commercial CS and P_1_A_3_@B‐MOF sponge were compared. Representative hemostatic images are shown in Figure 6H. The blood loss in the CS and P_1_A_3_@B‐MOF groups was 0.56 ± 0.04 g and 0.45 ± 0.03 g, respectively (Figure 6L), with bleeding times of 182 ± 8 and 154 ± 5 s (Figure 6K). The differences were statistically significant (*p* < 0.001).

The hemostatic efficacy of the P_1_A_3_@B‐MOF sponge was assessed in vivo using a rat femoral vein injury model. As depicted in Figure 6I, the P_1_A_3_@B‐MOF sponge group exhibited reduced blood loss (0.53 ± 0.10 g) compared to the P_1_A_3_ (0.6 ± 0.08 g) and CS groups (0.68 ± 0.14 g) (Figure 6M), demonstrating superior hemostatic performance over the commercial CS sponge. In addition, the P_1_A_3_@B‐MOF sponge achieved the shortest bleeding time of 192 ± 5 s (Figure 6N).

The hemostatic mechanism of the P_1_A_3_@B‐MOF sponge involves several critical processes: its porous structure rapidly absorbs blood and expands to fill the wound, concentrating clotting factors and promoting thrombus formation to create a physical barrier for hemostasis [54]. ZIF‐8 nanoparticles and aldehyde‐modified OPu increase surface roughness, activating platelet integrins (e.g., GPIIb/IIIa) to enhance adhesion and aggregation. The negatively charged collagen fibers within the PADM component further activate the intrinsic coagulation pathway (Factor XII → XI → IX → VIII) [55].

### 2.7 P_1_A_3_@B‐MOF Sponge Dressing was Applied to the Treatment of Diabetic Wounds in Rats

**FIGURE 7:**
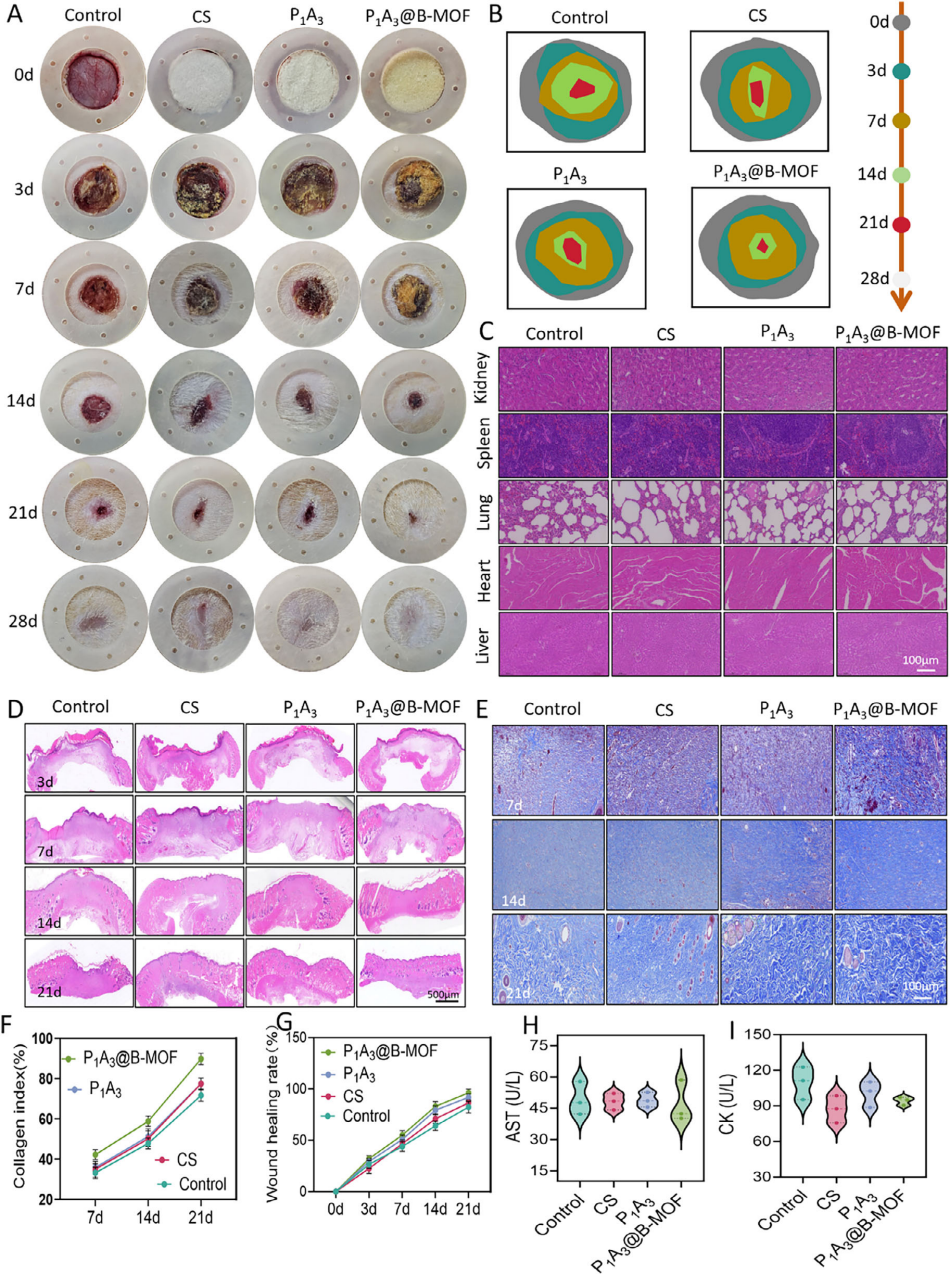
Treatment of diabetic wounds in rats using P_1_A_3_@B‐MOF sponge. (A) Representative images of the wound‐healing process. (B) Dynamic wound‐healing progression map. (C) H&E staining of the visceral organs. (D) H&E staining images. (E) Masson staining images. (F) Quantitative analysis of the collagen index. (G) Wound healing rate. (H) Quantitative analysis of AST levels in rat blood. (I) Quantitative analysis of CK in rat blood. Data (mean ± SD) were obtained from three independent experiments and analyzed using one‐way ANOVA with Tukey's post hoc multiple comparison test (*p < 0.05, **p < 0.01, ***p < 0.001).

In vivo evaluation of wound closure efficiency in diabetic rats revealed that the application of the P_1_A_3_@B‐MOF wound dressing significantly enhanced healing compared to that in the P_1_A_3_ and control groups. Photographic documentation demonstrated accelerated wound closure and epithelial regeneration in the P_1_A_3_@B‐MOF group, with nearly complete healing by day 21 and minimal scarring (Figure 7A,B). The wound closure rate reached 60 ± 10% on day 7 and 94 ± 6% by day 21, significantly outperforming P_1_A_3_ (87 ± 5%) and control groups (84 ± 6%) (Figure 7G). This superior healing outcome was primarily attributed to the effective release of SalB from the 3D sponge‐like structure of the P_1_A_3_@B‐MOF dressing, which facilitated the healing of diabetic wounds.

Histopathological analysis with hematoxylin and eosin (H&E) staining revealed significant neutrophil infiltration and pronounced tissue edema in the control group during the inflammatory phase (day 3). Conversely, the P_1_A_3_@B‐MOF group showed reduced inflammatory cell presence and uniform distribution, indicating that B‐MOF may alleviate chronic inflammation in diabetic wounds via anti‐inflammatory pathways. In the proliferative phase (days 7–14), the P_1_A_3_@B‐MOF group exhibited dense fibroblast proliferation, neovascularization, and notably thicker granulation tissue than the control group. During the remodeling phase (day 21), wounds treated with P_1_A_3_@B‐MOF displayed enhanced epithelial regeneration, healthier dermal components, gradual formation of hair follicles and sebaceous glands, and extensive neovascularization. By day 28, the P_1_A_3_@B‐MOF group exhibited lighter scar coloration (Figure 7D). As illustrated in Figure 7E, the collagen fibers in the P_1_A_3_@B‐MOF group were primarily organized in parallel bundles on day 7, whereas those in the control group were randomly distributed. The collagen index of the P_1_A_3_@B‐MOF group reached 58.5 ± 4.3% on day 14, which was approximately 1.3 times higher than that of the control group (43.5 ± 4.7%) (Figure 7F).

Serum biochemical tests and major visceral pathological staining were performed to assess the in vivo biocompatibility of the developed dressing. As shown in Figure 7C, the organs of the dressing group showed no significant cellular necrosis, fibrosis, or inflammatory responses compared to those of the control group. Liver function (AST) and kidney function (CK) test indicators revealed no statistically significant differences between the groups and the control group (Figure 7H,I). These findings preliminarily confirm the in vivo efficacy and safety of P_1_A_3_@B‐MOF, suggesting its potential for the treatment of diabetic wounds.

### 2.8 P_1_A_3_@B‐MOF Sponge Dressings Improve the Skin Immune Microenvironment and Promote Angiogenesis

**FIGURE 8:**
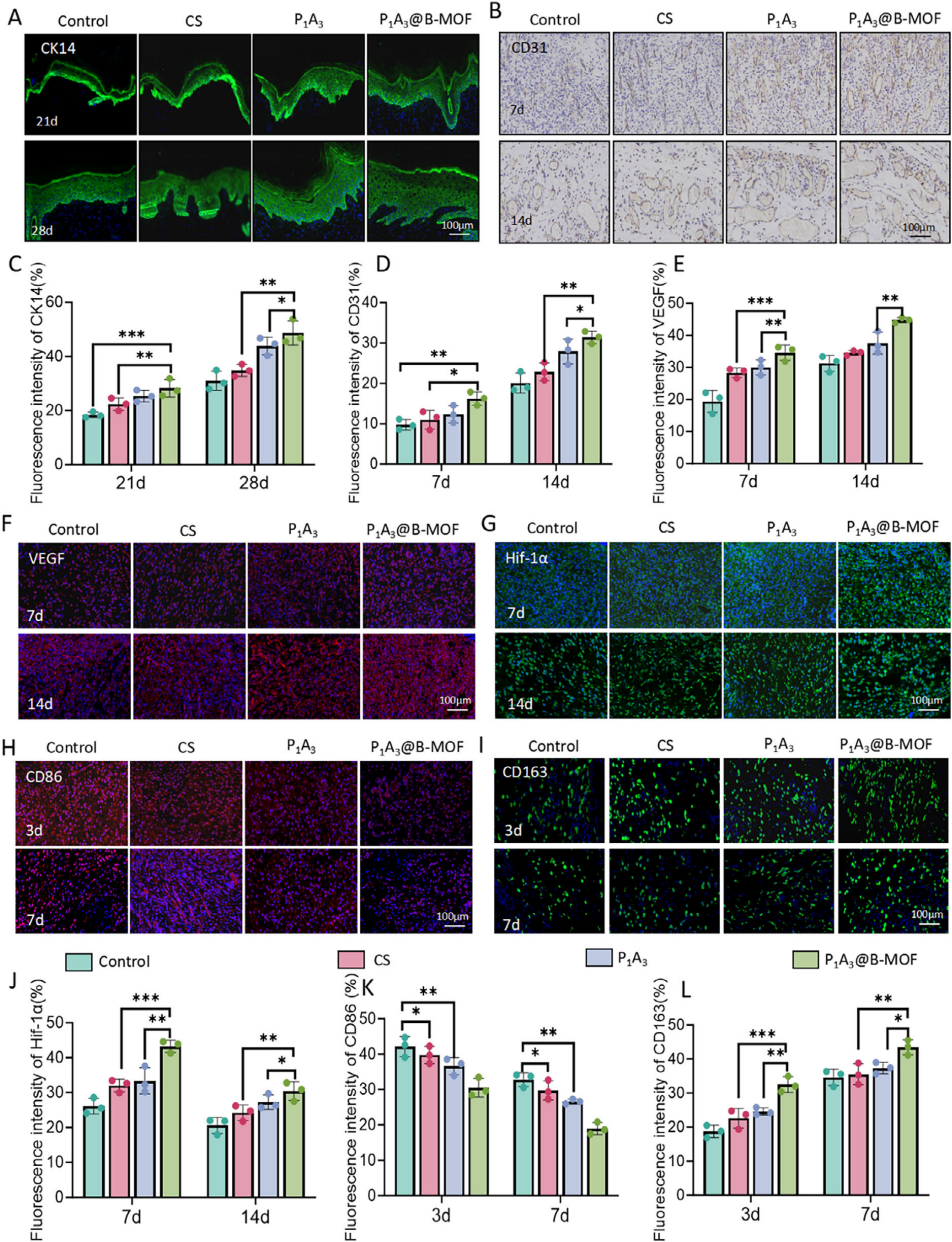
Immunohistological analysis. (A) Images of CK14 immunofluorescence staining. (B) Images of CD31 immunohistochemical staining. (C) Quantitative analysis of CK14 immunofluorescence. (D) Quantitative analysis of CD31 immunohistochemistry. (E) Quantitative analysis of VEGF immunofluorescence. (F) VEGF immunofluorescence staining images. (G) Images of Hif‐1α immunofluorescence staining. (H) Images of CD86 immunofluorescence staining. (I) Images of CD163 immunofluorescence staining. (J) Quantitative analysis of Hif‐1α immunofluorescence. (K) Quantitative analysis of CD86 immunofluorescence. (L) Quantitative analysis of CD163 immunofluorescence. Data (mean ± SD) were obtained from three independent experiments and analyzed using one‐way ANOVA with Tukey's post hoc multiple comparison test (*p < 0.05,** *p* < 0.01, ***p < 0.001).

CK14, a crucial biomarker for epidermal differentiation and epithelial regeneration, is present in hair follicles and epithelial cells. CK14 immunofluorescence staining was used to assess epithelial regeneration and follicular growth during wound healing. As shown in Figure 8A, green fluorescence intensified over time in all groups, with the P_1_A_3_ and P_1_A_3_@B‐MOF groups showing markedly stronger fluorescence (Figure 8C). By day 28, these groups exhibited distinct and continuous epidermal structures with no visible residual wounds. The P_1_A_3_@B‐MOF group exhibited the most complete and extensive epithelial coverage, whereas the control group did not achieve full epithelial coverage. Effective wound healing is closely associated with early local neovascularization [56].

CD31, a vital marker of vascular endothelial cells, is responsive to angiogenesis at the wound site. Immunohistochemical analysis (Figure 8B) and subsequent quantification (Figure 8D) revealed that the P_1_A_3_@B‐MOF group exhibited the highest density of CD31‐positive vessels, covering 35.2 ± 3.4% of the area. Hif‐1α, a pivotal transcription factor in hypoxia, binds to the HRE region of the VEGF promoter, significantly enhancing VEGF expression and playing a key role in angiogenesis. Immunofluorescence staining showed a progressive increase in VEGF (Figure 8F) and Hif‐1α (Figure 8G) expression across all groups from day 7 to day 14 of wound healing. Notably, B‐MOF significantly elevated VEGF and Hif‐1α expression (Figure 8E,J), facilitating early vascularization and aiding chronic wound healing. The enhanced angiogenesis in P_1_A_3_@B‐MOF is likely due to SalB's ability to stabilize Hif‐1α and boost its transcriptional activity, thereby upregulating the expression of VEGF. SalB also enhances VEGF expression via the IL‐10/STAT3 pathway. Moreover, SalB may inhibit PTEN to activate the PI3K/Akt pathway, thereby improving endothelial cell responses to VEGF and enhancing VEGFR2 signaling. In patients with diabetes, damaged skin tissues exhibit high levels of inflammatory signals such as ROS and glucose [57]. The loose tissue structure and pervasive cellular damage impede the immune microenvironment and hinder tissue repair processes.

CD86, a key costimulatory molecule on antigen‐presenting cells (APCs), including macrophages and dendritic cells, interacts with T‐cell markers CD28 or CTLA‐4 to modulate T‐cell activation and immune responses. Immunofluorescence on days 3 and 7 postsurgery revealed strong CD86 staining in the control group, followed by the CS group, with significantly reduced levels in the P_1_A_3_ and P_1_A_3_@B‐MOF groups (Figure 8H,K). CD163, a marker of M2 macrophage activation, mitigates excessive monocyte activation during infections and inflammation by decreasing proinflammatory cytokines, such as TNF‐α and IL‐1β, and reducing chemokines, such as MCP‐1, while enhancing anti‐inflammatory mediators, such as IL‐10 [58]. Figure 8I,L illustrate significantly higher CD163 levels in the P_1_A_3_@B‐MOF group than in the CS and P_1_A_3_ groups, indicating attenuated inflammatory responses. These results suggest that P_1_A_3_@B‐MOF exhibits strong anti‐inflammatory effects, likely due to Zn^2+^ activation of the STAT3 pathway, which boosts CD163 expression and M2 polarization and inhibits the NF‐κB pathway, reducing M1‐associated factors [59]. Collectively, P_1_A_3_@B‐MOF sponges significantly reduced wound inflammation and enhanced angiogenic factor expression, promoting vascular endothelial cell migration and proliferation.

### 2.9 P_1_A_3_@B‐MOF Sponge Dressings Reduce Scar Formation of the Wound

**FIGURE 9:**
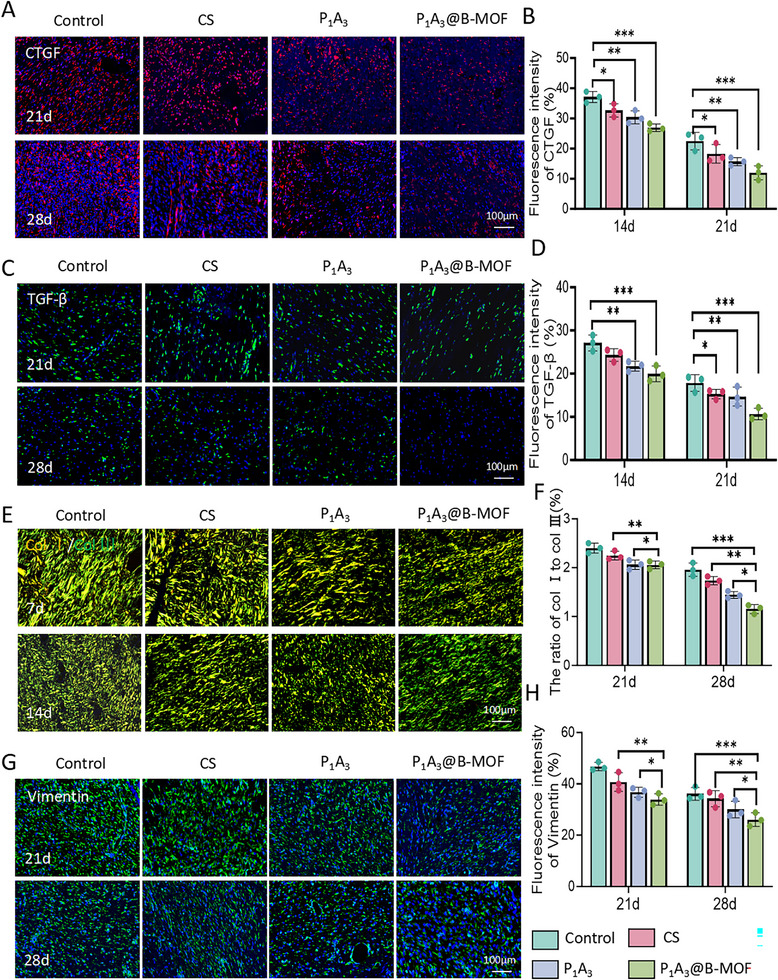
Immunohistological analysis. (A) Images of CTGF immunofluorescence staining. (B) Quantitative analysis of CTGF immunofluorescence intensity. (C) Images of TGF‐β immunofluorescence staining. (D) Quantitative analysis of TGF‐β immunofluorescence intensity. (E) Sirius red staining images. (F) Ratio of Col I to Col III. (G) Vimentin immunofluorescence images. (H) Quantitative analysis of vimentin immunofluorescence intensity. Data (mean ± SD) were derived from three independent experiments and analyzed using one‐way ANOVA with Tukey's post hoc test (*p < 0.05, **p < 0.01, ***p < 0.001).

We performed immunohistochemical staining to assess scar formation during the wound healing process. Connective tissue growth factor (CTGF), a critical component of the CCN family, significantly influences fibrosis and wound healing. Its expression, which is regulated by TGF‐β, mechanical stress, and hypoxia, is closely linked to extracellular matrix deposition, cell proliferation, and scar formation [60]. Figure 9A shows a time‐dependent decrease in CTGF (red) fluorescence across all groups. Quantitative fluorescence intensity analysis indicated the following trend: Control > CS > P_1_A_3_ > P_1_A_3_@B‐MOF (Figure 9B). Elevated CTGF expression suggests pathological scar formation, including hypertrophic scars and fibrosis. We propose that Zn^2+^ released by P_1_A_3_@B‐MOF may inhibit NF‐κB signaling to reduce inflammation‐related CTGF expression or modulate metalloproteinase (MMP) activity to curb excessive ECM deposition, thereby mitigating hyperplastic fibrosis.

TGF‐β, a multifunctional cytokine superfamily, regulates cell growth, differentiation, immune responses, and extracellular matrix remodeling, and plays a crucial role in tissue repair, fibrosis, and immune regulation [61]. As shown in Figure 9C, the TGF‐β (green) fluorescence area decreased over time in all the groups. Quantitative fluorescence intensity analysis revealed that the P_1_A_3_@B‐MOF group exhibited the weakest fluorescence (Figure 9D). TGF‐β activates CTGF upstream, creating a positive feedback loop that leads to ECM deposition and promotes the synthesis of collagen types I and III as well as fibronectin. In the later stages of wound healing, persistent TGF‐β activation leads to fibrosis and scar formation. The P_1_A_3_@B‐MOF group showed the lowest TGF‐β expression, likely due to SalB release, which inhibits Smad2/3 phosphorylation, decreases type I collagen/CTGF expression, and increases Smad7 (an inhibitory Smad), thus establishing a negative feedback loop that suppresses TGF‐β signaling [62].

Sirius red is an acidic dye that can combine with the basic amino acids in collagen fibers, such as hydroxyproline. Under a polarized light microscope, it shows a birefringence phenomenon (type I collagen appears orange/red and type III collagen appears green). Sirius red staining was used to evaluate collagen I and III levels in the tissue. Further analysis (Figure 9E) showed that the proportion of collagen types in the P_1_A_3_@B‐MOF group was balanced, regularly arranged in bundles or reticulates, and evenly distributed. Type I collagen (red–orange) was slightly more than type III collagen (green). In the control group, the proportion of type I/type III collagen increased and was gradually replaced by thicker type I collagen (Figure 9F), suggesting a risk of hypertrophic scar hyperplasia.

Vimentin, an essential intermediate filament protein in the cytoskeleton, is pivotal in mesenchymal‐derived cells, such as fibroblasts, endothelial cells, and immune cells. Activated fibroblasts, or myofibroblasts, express high levels of vimentin and secrete substantial ECM, including type I collagen, which facilitates scar contraction [63]. As illustrated in Figure 9G, vimentin expression peaked on day 21 and then declined during the wound healing process. The P_1_A_3_@B‐MOF group showed the lowest vimentin expression compared to the P_1_A_3_ and control groups (Figure 9H). This reduction is primarily attributed to the sustained release of B‐MOF, which inhibits TGF‐β/Smad3 signaling, indirectly downregulating vimentin, and limiting myofibroblast transformation. These immunofluorescence studies conclusively demonstrated that P_1_A_3_@B‐MOF effectively reduced wound scar formation by disrupting the TGF‐β‐CTGF feedback loop, thereby decreasing collagen deposition, suppressing chronic inflammation, and inhibiting fibrosis [64].

### 2.10 Potential Mechanisms and Limitations

This study successfully developed and validated an in situ embedded B‐MOF sponge with shape‐memory functionality (P_1_A_3_@B‐MOF) as an integrated dressing for diabetic wound therapy, offering an innovative spatiotemporally controlled combination strategy. The core therapeutic mechanisms and value are reflected in the following aspects: the design innovation lies in encapsulating SalB within the ZIF‐8 framework to construct a pH‐responsive intelligent drug delivery system. In vitro release profiles confirmed that the dressing rapidly released SalB and Zn^2+^ under a simulated acidic diabetic wound environment (pH = 5.4) while maintaining sustained release under physiological neutral conditions (pH = 7.4). This property enables targeted drug delivery at the pathological site, significantly improving the bioavailability of SalB and overcoming its limitations of short half‐life and rapid metabolism when applied directly. The highly porous structure of the sponge (porosity > 87%) allows rapid exudate absorption, and combined with its pH‐responsive drug release behavior, facilitates the targeted delivery of SalB and Zn^2+^ in the acidic wound microenvironment, forming a spatiotemporally controlled therapeutic advantage. Its shape‐memory property further enables adaptation to irregular wounds, providing continuous protection. The Zn^2+^ released from B‐MOF degradation inhibits NF‐κB activity, reduces the expression of proinflammatory factors such as TNF‐α, and promotes macrophage polarization from the M1 to M2 phenotype. Additionally, B‐MOF upregulates the Hif‐1α/VEGF signaling pathway, leveraging and guiding hypoxic signals in the wound to actively drive angiogenesis, thereby addressing the fundamental issue of nutrient supply. In a diabetic rat model, P_1_A_3_@B‐MOF accelerated wound closure (achieving a 98.5% healing rate by day 14) and downregulated the TGF‐β/Smad signaling pathway, reducing CTGF expression and interrupting the TGF‐β → CTGF → collagen deposition feedback loop. It balanced the ratio of type I/III collagen (as shown by more organized collagen arrangement in Sirius red staining) and suppressed myofibroblast overactivation and vimentin expression, effectively inhibiting scar formation and achieving simultaneous functional and aesthetic recovery.

Although this study has demonstrated the significant potential of the composite dressing in treating acute and chronic wounds, there are several key limitations that must be addressed before transitioning to clinical applications. First, at the biological mechanism level, while we have confirmed that Zn^2+^ and SalB can synergistically regulate macrophage polarization, the precise signaling networks downstream and the cascade interaction mechanisms among immune cells remain to be elucidated. Second, from a material and safety perspective, the adhesive properties and mechanical performance of the current dressing require optimization for long‐term applicability in dynamic joint areas, and there is a lack of direct comparative data with existing clinical standard dressings [65]. Moreover, the long‐term release kinetics of SalB, the in vivo metabolic pathways of Zn^2+^, and their potential cumulative toxicity have not been systematically evaluated in large animal models or over extended observation periods. This represents a critical safety concern that must be addressed before advancing to preclinical research. Future work will focus on these identified limitations: in‐depth analysis of immune regulatory networks through proteomics, genetic manipulation, and multicellular coculture models. Additionally, material mechanical properties will be optimized, and comprehensive preclinical safety evaluations will be conducted to facilitate the transition to clinical applications.

## 3 Conclusions

In summary, we developed an in situ B‐MOF sponge dressing (P_1_A_3_@B‐MOF) for comprehensive wound management of diabetic wounds. Using a one‐pot synthesis approach, B‐MOF was synthesized on a P_1_A_3_ sponge, endowing the P_1_A_3_@B‐MOF dressing with angiogenic, immune‐regulating, and active exudate management capabilities. In vitro experiments demonstrated that the sponge modulated macrophage polarization, reducing oxidative stress and immune dysregulation, and promotes the migration and angiogenesis of HUVECs. In rat hemorrhage models, P_1_A_3_@B‐MOF demonstrated rapid hemostatic capability by rapidly absorbing blood, swelling to fill the wound, and concentrating coagulation factors, thereby enhancing the hemostatic efficacy. Furthermore, in diabetic rat models, P_1_A_3_@B‐MOF attenuated inflammatory responses, stimulated granulation tissue formation and angiogenesis, promoted reepithelialization and scarless skin regeneration, and suppressed scar formation. This sponge dressing provides a promising therapeutic strategy for comprehensive diabetic wound management and scar‐reduced healing, paving the way for customized biomaterials in soft tissue injury repair and various reconstructive scenarios.

## 4 Materials and Methods

### 4.1 Materials

Pullulan (9057‐02‐7, Macklin), sodium periodate (7790‐28‐5, Macklin), methanol (67‐56‐1,Macklin), 2‐methylimidazole (1739‐84‐0, Macklin), Zn(NO_3_)_2_·6H_2_O, glutaraldehyde (P890356, Macklin), paraformaldehyde (P890358, Macklin), collagenase type I (9001‐12‐1, Macklin), and SalB (121521‐90‐2, Macklin) were all purchased from Macklin (Shanghai, China).The Live/Dead Staining Kit (Calcein‐AM/PI, U23‐002A) was purchased from YOBIBIO. Matrigel (M8380) was obtained from Solarbio. Phalloidin (iFluor 488, CA1610) and 4’,6‐diamidino‐2‐phenylindole (DAPI, ZRXX13528) were from zrbiorise. Anti‐VEGF antibody (Product # M808) / anti‐Hif‐1α antibody (Product # 700 505), Mouse secondary antibody (Product # A‐11008, 1:500), IL‐13 (MBNPM‐0454‐A, Maicikelin), TNF‐α (Product # MA5‐23720) / IL‐10 (Product # MA5‐42656) / CD86 (Product # MA5‐48134) / CD163 (Product # MA5‐54106), Crystal Violet (CA0059, MAIGE), Bacterial Live/Dead Staining Kit (DMAO/PI, C2030S, Beyotime), TGF‐β (Product # MA1‐21595), Vimentin (Product # MA5‐35320), and CK14 (Product # KRTL/4440R). The collagen hemostatic sponge was purchased from Wuxi Beidi Bioengineering Co., Ltd. (Jiangsu, China). Immortalized HUVECs (LH‐H089) and immortalized human epidermal keratinocytes (HaCaT) (nobcell0075) were obtained from Baiha Biotechnology and Nuobo Biological, respectively.

### 4.2 Preparation of Oxidized Pullulan Polysaccharide‐Decellular Dermal Matrix Sponges

Pullulan (5 g) was dissolved in deionized water (500 mL) to prepare a 1% w/v solution. Subsequently, sodium periodate (NaIO_4_, 0.5 g) was added, and the mixture was stirred in the dark for 6 h. Ethylene glycol was added to terminate the reaction, and the mixture was stirred for 2 h. The mixture was dialyzed for 3 d, freeze‐dried, and stored in the dark, resulting in a product called OPu. The PADM was prepared according to a previously described protocol [66]. In this study, 5%OPu was mixed with 1.5% PADM solution in a volume ratio of 1:1, 1:3, 1:5, 3:1, 5:1, and 1:5, respectively. The mixture was then poured into molds and lyophilized to form sponges, which were named P_1_A_1_, P_1_A_3_, P_1_A_5_, P_3_A_1_, and P_5_A_1_.

### 4.3 Preparation of P_1_A_3_@B‐MOF Sponge

A 0.594 g sample of Zn(NO_3_)_2_·6H_2_O and 0.1 g of SalB were dissolved in 20 mL of methanol and magnetically stirred until complete dissolution (solution A). Separately, 1.313 g of 2‐methylimidazole was dissolved in 20 mL of methanol and stirred until the solution became clear (solution B). The vacuum‐degassed P_1_A_3_ sponge was first immersed in solution A and stirred slowly at 25°C and 100 rpm for 6 h. After removing the sponge, it was quickly transferred to solution B, and then stirred slowly at 25°C for 6 h. The sponge was washed 3 times with methanol to remove unreacted substances, and then lyophilized for 48 h to obtain the P_1_A_3_@B‐MOF sponge.

### 4.4 Characterization of Physical and Chemical Properties of Materials

The sponge samples were trimmed, mounted on a sample stage using a conductive adhesive, and sputter‐coated with gold (60 s). The samples were then examined using a scanning electron microscope (Regulus 810, Hitachi, Japan) for morphological analysis. The elemental composition was assessed using an Oxford EDS attached to the SEM. The Pu/OPu and MOF samples were mounted on silicon wafer substrates for XRD analysis using a SmartLab SE Rigaku Japan instrument, with scanning angles from 5° to 80° (2*θ*) at 5°/min to determine their crystal structures. Elemental analysis and binding energy measurements of the prepared P_1_A_3_ sponge were conducted using a Thermo ESCELAB 250XI X‐ray photoelectron spectrometer. FTIR spectra were recorded using a Nicolet iS50 FTIR spectrometer (Thermo, USA) over a range of 4000–500 cm^−1^. The lyophilized OPu samples were vacuum‐dried at 50°C for 24 h. Approximately 5 mg of the sample was dissolved in 0.6 mL of DMSO‐d_6_, and the mixture was incubated at room temperature under shaking overnight before testing. The NMR spectrum was acquired using a 400 MHz NMR spectrometer at 25°C, with a spectral width of 12 ppm, relaxation delay of 2.0 s, and 64 accumulations. The characteristic peak shift was analyzed using the residual proton peak of DMSO‐d_6_ (*δ* = 2.50 ppm) as an internal standard.

The hydrophobic and hydrophilic properties of the sponge samples were assessed and photographed using a Water Contact Angle Tester (Theta Flex Biolin, Finland). Porosity, water absorption, and density were measured using a porosity tester for polyceramic materials (JHY‐120C). A tensile testing machine (DR‐603A) was used to perform 15 compression cycles on the sponge at a rate of 10 mm/min, achieving 60% deformation per cycle.

The resulting curves were recorded, and the compression modulus was calculated. The sponge samples were fabricated into dry cylindrical specimens with a diameter of 12 mm and a height of 14 mm. Deionized water was then dripped onto the sponges and uniform pressure was applied. The morphologies of the samples were documented and photographed before, during, and after compression.

### 4.5 Drug Release Experiment

To evaluate the release kinetics of SalB from the P_1_A_3_@B‐MOF construct, 100 mg of the material was dispersed in 50 mL of release medium. The suspension was transferred to a pretreated dialysis bag and immersed in 50 mL of buffer solution at pH 5.4 or 7.4. The dialysis bag was placed in a 37°C water bath and shaken at 100 rpm. At predetermined time points, 1 mL of the sample was withdrawn, and the buffer was replenished with a fresh isothermal solution to maintain the volume. The concentration of SalB in the samples was determined using high‐performance liquid chromatography (HPLC). The cumulative release was calculated as follows: Cumulative Release Rate (%) = (*M*_t_/*M*_L_) × 100, where *M*_t_ is the cumulative amount of drug released at time t, and *M*_L_ is the total amount of drug loaded in ZIF‐8.

For the Zn^2+^ release experiment, 100 mg samples of P_1_A_3_@B‐MOF were presoaked in the appropriate pH buffer (pH = 5.4 or 7.4) for 5 min to reach swelling equilibrium. The treated samples were transferred to 50 mL centrifuge tubes, 20 mL of preheated buffer solution (37°C) was added, and the samples were incubated in a 37°C water bath with constant agitation at 100 rpm. At each time point, 1 mL of supernatant was collected, filtered through a 0.22 µm filter, and immediately mixed with 9 mL of a 1% HNO_3_ acidification buffer solution. The system was replenished with a fresh buffer at the same temperature to maintain its volume. Three parallel experiments (*n* = 3) were conducted, and the ion concentrations were measured using an inductively coupled plasma optical emission spectrometer (ICP‐OES). The cumulative release of Zn^2+^ (*Q_t_*) was calculated using the formula *Q_t_* = *C_n_* × *V* + Σ (*C_i_* × *V_s_*), where *C_n_* is the concentration at the *n*th measurement, *V* is the system volume, *C_i_* is the concentration at the *i*‐th sampling point, and *V_s_* is the sampling volume. Each experimental group was subjected to three independent repetitions (*n* = 3).

### 4.6 Biocompatibility Evaluation

The cytotoxicity of the sponge samples was evaluated using a Live/Dead Cell Viability/Cytotoxicity Assay Kit (Calcein‐AM/PI, U23‐002A, YOBIBIO). HUVECs in the logarithmic growth phase were seeded in 96‐well plates at a density of 1 × 10^4^ cells per well. The cells were treated with sponge extract for 24, 48, and 72 h. Following incubation, the cells were stained with calcein‐AM (for live cells, green fluorescence) and Propidium Iodide (PI, for dead cells, red fluorescence) for 30 min at 37°C. Cell viability was assessed by observing live (green) and dead (red) cells under an inverted fluorescence microscope (Nikon Ti‐S). Each experimental group was subjected to 3 times independent repetitions (*n* = 3).

### 4.7 Cell Migration and Tubule Formation Experiments

HUVECs were seeded in 6‐well plates and cultured until they reached approximately 80% confluence. A scratch was created using a 200 µL pipette tip. The experimental group was treated with Dulbecco's modified Eagle Medium (DMEM) containing 5 mg/mL sponge extract (2% serum), whereas the control group was treated with DMEM containing only 2% serum. Images were captured at 0, 24, and 48 h using an inverted fluorescence microscope (Nikon Ti‐S). Cell migration was quantified using ImageJ by comparing the initial scratch area (*W*_0_) to the area at each time point, calculated as follows: Migration Rate = ((*W*_0_ − *W*_t_)/*W*_0_) × 100.

In a separate experiment, HUVECs were seeded in 6‐well plates and grown to 50% confluence, followed by incubation with DMEM containing 5 mg/mL sponge extract for 24 h. The thawed matrix gel was distributed into 96‐well plates, and HUVECs (50,000 cells per well) were inoculated onto the solidified gel (M8380, Solarbio). After 12 h of coculture, the cells were stained using a live/dead dye kit (Calcein‐AM/PI) for 20 min. Fluorescence microscopy (Nikon Ti‐S) was used to image vascular formation, which was analyzed using the ImageJ software. Each experimental group was subjected to 3 times independent repetitions (*n* = 3).

### 4.8 Cell Adhesion Experiment

HUVECs in the logarithmic growth phase were digested and suspended at a concentration of 5 × 10^5^ cells per mL, and then inoculated for a 72 h coculture. Immunofluorescent staining was performed using iFluor 488 (CA1610,Solarbio) and DAPI (ZRXX13528, zrbiorise), and images were acquired using laser‐scanning confocal microscopy (ZEISS/LSM 980). Each experimental group was subjected to 3 times independent repetitions (*n* = 3).

### 4.9 Cellular Immunofluorescence Staining

HUVECs were seeded (1 × 10^4^ cells per slide) and cultured for 72 h using the sponge extract. The cells were then fixed with 4% paraformaldehyde, permeabilized with 0.1% Triton X‐100, and incubated overnight at 4°C with primary antibodies against VEGF (Product # M808, 1:100) and HIf‐1α (Product#700 505, 1:200). After incubation with the secondary antibody (Product#A‐11008, 1:500) in the dark for 1 h, the nuclei were stained with DAPI. Images were acquired using a laser confocal microscope (ZEISS LSM 980). Each experimental group was subjected to 3 times independent repetitions (*n* = 3).

### 4.10 Evaluation of Macrophage Polarization and Anti‐Inflammatory Activity

Using the murine macrophage cell line RAW 264.7 as a model, we evaluated the effects of sponge dressing on macrophage polarization and anti‐inflammatory activity. RAW 264.7 cells (5 × 10^4^ cells per well) were cultured in standard plates and on plates suitable for confocal microscopy, as described above. After 12 h of incubation, the cells were washed with phosphate‐buffered saline (PBS). The positive control group received either 500 ng/mL LPS (93572‐42‐0, Aladdin) or a mixture of 20 ng/mL IL‐4(P5916, Beyotime) and 20 ng/mL IL‐13(MBNPM‐0454‐A, Maicikelin) in blank medium. The experimental group was treated with a 500 ng/mL LPS solution or a 20 ng/mL IL‐4 and 20 ng/mL IL‐13 mixture, both combined with 10 mg/mL sponge extract. The negative control group contained only blank medium. After 36 h of culture, the cells were rinsed with PBS, fixed with 4% paraformaldehyde, and permeabilized. Nonspecific binding sites were blocked with 1% bovine serum albumin for 30 min. For RAW 264.7 cells, TNF‐α (Product # MA5‐23720), IL‐10 (Product # MA5‐42656), CD86 (Product # MA5‐48134), and CD163 (Product # MA5‐54106) were used as primary antibodies. The fluorescently labeled stained cells were imaged using a laser scanning confocal microscope (ZEISS/LSM 980). Each experimental group was subjected to 3 times independent repetitions (*n* = 3).

### 4.11 Cell Invasion Assay

A Transwell migration assay was employed to evaluate the effect of sponge materials on the migration ability of HUVECs. The specific procedure was as follows: After serum starvation for 12 h, HUVECs were resuspended in serum‐free medium at a density of 2 × 10^4^ cells per 200 µL and seeded into the upper chamber of a Transwell insert. The sponge material was placed in the lower chamber of the Transwell. The system was incubated at 37°C under 5% CO_2_ for 48 h. After incubation, nonmigrated cells in the upper chamber were removed using a cotton swab. Cells that migrated to the lower chamber were fixed with 4% paraformaldehyde for 20 min and stained with 0.1% crystal violet (CA0059, MAIGE) for 15 min before being imaged. The number of migrated cells was observed and recorded using an Olympus optical microscope. A quantitative analysis was performed to assess the effects of different sponge materials on HUVEC migration. Each experimental group was subjected to 3 times independent repetitions (*n* = 3).

### 4.12 Cell Infiltration and Adhesion

HUVECs were cocultured for 3 d in 96‐well plates containing sterile sponge scaffold extracts at a density of 3.0 × 10^4^ cells per scaffold. DAPI/phalloidin fluorescence staining was performed to observe cell infiltration and adhesion within the scaffolds.

### 4.13 Blood Compatibility Evaluation

Rat red blood cells (RBCs) were isolated from whole blood mixed with sodium citrate (9:1) and washed with PBS to prepare a 5% v/v cell suspension for flow cytometric analysis. The composite dressing powder was generated by grinding the lyophilized sample to obtain a 2.5 mg/mL powder dispersion. The RBC suspension was mixed with the composite dispersion at varying concentrations, incubated at 37°C for 1 h, and centrifuged at 10,000 rpm for 10 min. The absorbance of the supernatant was measured at 562 nm using a microplate reader, with deionized water and PBS serving as positive and negative controls, respectively. Hemolysis percentage was calculated as: Hemolysis (%) = (A_m_ − A_p_)/(A_h_ −A_p_) × 100%, where A_m_ represents the sample supernatant, A_h_ is the absorbance of the positive control (water), and A_p_ is the absorbance of the negative control (PBS). Each experimental group was subjected to 3 times independent repetitions (*n* = 3).

### 4.14 Evaluation of Antibacterial Performance of P_1_A_3_@B‐MOF Sponge Using Coculture Method

To assess the in vitro antibacterial activity of P_1_A_3_@B‐MOF, a coculture determination method was employed. The sponge samples were placed in a 24‐well plate containing a bacterial suspension with a concentration of 1 × 10^8^ CFU/mL, with 1 mL of the bacterial solution added to each well. The 24‐well plate was then incubated at 37°C for 24 h. After incubation, the culture medium was removed, and the surface of the material was gently rinsed with PBS to collect any residual bacteria, which was then combined with the original culture medium. Subsequently, the bacterial solution was diluted 10^5^‐fold, and 100 µL of each dilution was evenly spread on the surface of blood agar plates, which were then inverted and incubated at 37°C for an additional 24 h. After the incubation period, the blood agar plates were subjected to colony counting and photographed for records. Three parallel replicates were performed for each experiment, with wells containing no added material serving as a blank control, and the antibacterial rate was calculated.

### 4.15 Evaluation of Antibacterial Performance of P_1_A_3_@B‐MOF Sponge Using Live/Dead Bacterial Staining

To further quantify the antibacterial effect of the P_1_A_3_@B‐MOF sponge material, a live/dead bacterial staining method was employed. The dressing samples were cocultured with 1 mL of bacterial suspension (1 × 10^8^ CFU/mL) in a 24‐well plate at 37°C for 24 h. After incubation, the bacterial suspension was collected, and centrifuged at 8,000 rpm for 5 min to discard the supernatant. A live/dead bacterial staining kit (DMAO/PI, Biyuntian, C2030S) was used for staining: the bacterial pellet was resuspended in PBS, followed by the addition of DMAO dye (final concentration 5 µm) and PI dye (final concentration 10 µm) and incubated in the dark for 15 min. After staining, the bacteria were washed 3 times with PBS (8,000 rpm, 5 min) to completely remove any unbound dye. Finally, the bacterial pellet was resuspended in 100 µL PBS, and 10 µL of the bacterial solution was placed on a glass slide, covered with a cover slip, and then observed using a Nikon ECLIPSE Ti inverted fluorescence microscope. Green fluorescence indicated the presence of all bacteria, while red fluorescence indicated dead bacteria. Five random fields of view were selected for counting each sample, with the experiment repeated 3 times. The relative antibacterial rate of the sponge was quantified by calculating the ratio of red to green fluorescent bacteria.

### 4.16 Coagulation Time Determination

500 mg of each sponge sample was placed into a test tube, and 2 mL of activated whole rat blood (containing 50 µL of 0.2 m CaCl_2_) was added. Blood flow was observed by inverting the test tube every 15 s, and the time until blood flow ceased was recorded as coagulation time. Each experimental group was subjected to 3 times independent repetitions (*n* = 3).

### 4.17 BCI Determination

A cylindrical hemostatic sponge (10 mm diameter, 10 mm height) was incubated at 37°C in a Petri dish with 1 mL of citrated rat blood (3.8% sodium citrate) and 20 µL of CaCl_2_ solution (0.2 m). Following the addition of 25 mL of distilled water, the mixture was incubated at 37°C for 5 min. The dishes were then placed on a fixed rhythm shaker and gently shaken for 5 min. The absorbance of the supernatant was measured at 545 nm using a Thermo Varioskan ALF multiwell plate reader, with a control sample containing 200 µL of blood and 25 mL of deionized water. The BCI was calculated as follows: BCI (%) = (OD_Sample_/OD_Control_) × 100%, where OD_Sample_ and OD_Control_ represent the absorbance values at 545 nm for the sample and control groups, respectively. Each experimental group was subjected to 3 times independent repetitions (*n* = 3).

### 4.18 Blood Cell Adhesion Experiment

RBCs were incubated with the sponge at 37°C for 30 min, after which the unattached cells were removed by washing with PBS. The samples were then fixed in 2.5% glutaraldehyde at 4°C for 2 h, followed by dehydration using a graded series of ethanol (30%, 50%, 70%, 80%, 90%, 95%, and 100% v/v). After drying at room temperature and gold sputter coating, the number of adherent blood cells was observed using scanning electron microscopy. Each experimental group was subjected to 3 times independent repetitions (*n* = 3).

### 4.19 Rat Femoral Vein Injury Model

Animal experiments were performed in accordance with the National Research Council's Guide for the Care and Use of Laboratory Animals. Rats were anesthetized with 2% sodium pentobarbital, the groin hair was shaved, and an incision was made in the groin to expose the femoral vessel. A 20‐G needle was used to puncture the femoral vein, and the wound was allowed to bleed freely for 5 s. A sponge was then applied to the wound and manually compressed using preweighed medical gauze. Hemostasis time was measured from the time of sponge application until bleeding ceased. Blood loss was determined by weighing the gauze and sponges. Each experimental group was subjected to 3 times independent repetitions (*n* = 3).

### 4.20 Rat Liver Defect Model

After anesthetizing the rats, the abdominal hair was removed, and the abdominal cavity was opened to expose the liver. The surrounding body fluid in the liver area was carefully drained, and preweighed filter paper was placed under the organs. A sterile biopsy punch was used to create a 3 mm diameter bleeding wound in the liver. Spontaneous bleeding was allowed for 10 s to ensure normal hemorrhage, after which a precompressed sponge was immediately applied to the wound to stop the bleeding. Blood loss was measured by weighing the filter paper beneath the liver, and the hemostasis time was recorded. Each experimental group was subjected to 3 times independent repetitions (*n* = 3).

### 4.21 Rat Tail Bleeding Model

We assessed the hemostatic efficacy of the sponges using a rat tail hemorrhage model. Following anesthesia, a premeasured filter paper was positioned under the tail, which was then transected 3 cm from its tip. The sponges were applied directly to the bleeding sites without pressure. Hemostasis time, blood loss volume, and clotting time were evaluated. Each experimental group was subjected to 3 times independent repetitions (*n* = 3).

### 4.22 Wound Healing in Diabetic Rats

Rats were intraperitoneally injected daily with 55 mg/kg of 1% streptozotocin (STZ, 18883‐66‐4, Macklin) solution. One week later, the rats displayed polydipsia, polyphagia, and polyuria, with random blood glucose levels exceeding 16.7 mm, confirming the induction of type I diabetes. Following intraperitoneal administration of 2% sodium pentobarbital for anesthesia, the dorsal skin was prepared and disinfected, and two full‐thickness cortical defects with a diameter of 12 mm were surgically created.

The wound sites were then treated with P_1_A_3_ sponge, P_1_A_3_@B‐MOF sponge, or Collagen sponge (CS) sponge dressings, respectively. No dressing was applied in the control group. Subsequently, all wounds were covered and secured with sterile gauze. Each experimental group included three rats (*n* = 3). If a sponge fell off, it was immediately replaced. The wound closure rate was calculated at multiple postoperative time points using the following formula: closure rate (%) = (*S*_0_ − *S_t_*)/*S*_0_ × 100%, where *S*_0_ represents the initial wound area and *S_t_* denotes the wound area recorded on each assessment day. Rat venous blood was collected into anticoagulant tubes, and hepatic and renal function parameters and complete blood counts were measured using an automated blood biochemistry analyzer. Tissue samples were collected at each time point. At 28 days postinjury, the heart, liver, spleen, lungs, and kidneys were excised, fixed in 4% paraformaldehyde, and embedded in paraffin blocks. Tissue sections were stained with H&E and Masson's trichrome and examined microscopically to analyze wound‐related changes. Collagen content was assessed using Sirius Red staining and polarized light microscopy. Each experimental group was subjected to 3 times independent repetitions (*n* = 3).

### 4.23 Histological Analysis

The tissue samples were fixed in 4% paraformaldehyde, paraffin‐embedded, and sectioned into 4 µm thick slices. Immunohistochemical staining was then performed. Immunohistochemistry or immunofluorescence staining was used to detect the expression of CD86, CD163, TNF‐α, IL‐10, VEGF, HIF‐1α, CK14 (Product # KRTL/4440R, 1:100), TGF‐β, CD31, CTGF, TGF‐β (Product # MA1‐21595, 1:100), and Vimentin (Product # MA5‐35320) in the wound tissues. Each experimental group was subjected to 3 times independent repetitions (*n* = 3).

### 4.24 Statistical Analysis

Data are presented as mean ± standard deviation. Statistical analyses were performed using Origin 2024, GraphPad Prism 10.1, and SPSS software (version 22.0). Unpaired *t*‐tests were used to compare two groups, whereas one‐way analysis of variance (ANOVA) was used for comparisons involving more than two groups. Statistical significance was set as follows: *p < 0.05, **p < 0.01, and ***p < 0.001.

## Author Contributions

**H.Z**.: writing – review & editing, methodology, investigation, data curation. **C.Y.H, Y.Q.C**.: writing – review & editing, methodology, investigation. **T.Z.Z**.: investigation, data curation, conceptualization. **F.Y.Z**.: funding acquisition, conceptualization. **G.J**.: Data curation and conceptualization. **J.R.Z**.: methodology, supervision, conceptualization. **Y.S.P**.: investigation, data curation, conceptualization. **Q.L.W**.: supervision, methodology, funding acquisition. **H.H.Z**.: supervision, methodology, funding acquisition, conceptualization. **L.Y, L.LC**.: writing – review & editing, supervision, methodology, funding acquisition, conceptualization.

## Ethics Statement

This study employed male Sprague Dawley (SD) rats (8‐10 weeks of age, weighing 220 ± 10 g), purchased from Zhuhai Bestway Biotechnology Co., Ltd. (License No. SCXK (Yue) 2020‐051). The experimental protocol (IACUC‐LAC‐20241225‐002‐2) was reviewed and approved by the Laboratory Animal Welfare Ethics Committee of Nanfang Hospital, Southern Medical University. All experimental procedures complied with the Guidelines for the Care and Use of Laboratory Animals and the ethical standards outlined by the National Natural Science Foundation of China. The animals were housed in a controlled environment at 21°C with a 12 h light‐dark cycle, acclimated for 7 days, and provided with free access to water and standard rodent feed.

## Conflicts of Interest

The authors declare no conflicts of interest.

## Supporting information




**Supporting File**: adhm70808‐sup‐0001‐SuppMat.docx.

## Data Availability

The data that support the findings of this study are available in the supplementary material of this article.
