# Supplementary material for: An In Situ Embedded B‐MOF Sponge With Shape‐Memory for All‐in‐One Diabetic Wound Therapy
Source: Adv Healthc Mater. 2026 Jan 22;15(14):e05350. doi: 10.1002/adhm.202505350 (PMC13068297; doi:10.1002/adhm.202505350)
Supplement: Supplementary file 1 — Supporting File: adhm70808‐sup‐0001‐SuppMat.docx. [file ADHM-15-0-s001.docx]

**An In-Situ Embedded B-MOF Sponge with Shape-Memory for All-in-One Diabetic Wound Therapy**

Hai Zhou^1,†^,Chaoyang Huang^2,†^, Yanqi Chen^2,†^, Tingzi Zhao^2,†^, FengYi Zhu^1^, Gong Jun^1^, Jinrong Zhang^1^, Yingsong Pan^1^, Qiulan Wen^3,*^, Lei Yang^2,*^, HuiHui Zhang^2,*^, Lianglong Chen^1,*^

^1^ Central Laboratory of Yunfu People's Hospital, Yunfu People's Hospital, Yunfu City, 527399, PR China

^2^Department of Burns, Nanfang Hospital, Southern Medical University, Jingxi Street, Baiyun District, Guangdong, 510515, PR China

^3^Department of Orthopaedic Surgery, Nanfang Hospital, Southern Medical University, Guangzhou, Guangdong, People's Republic of China.

^†^ These authors contributed equally to this study.

* Corresponding author.

E-mail: cllwql@163.com (L. Chen).


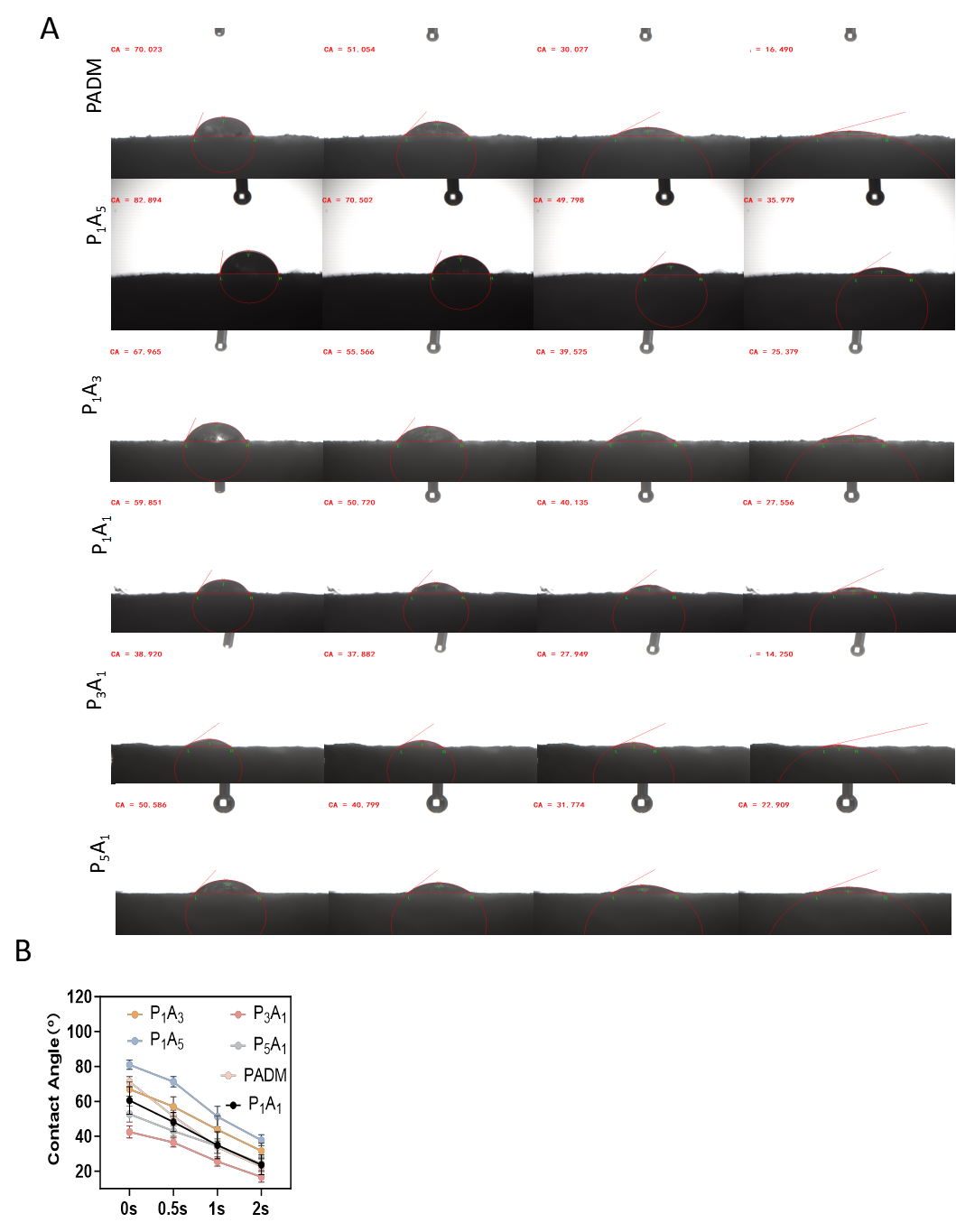


**Figure S1. Hydrophilicity of sponges.** (A) Water contact angle image. (B) Quantification of water contact angle.


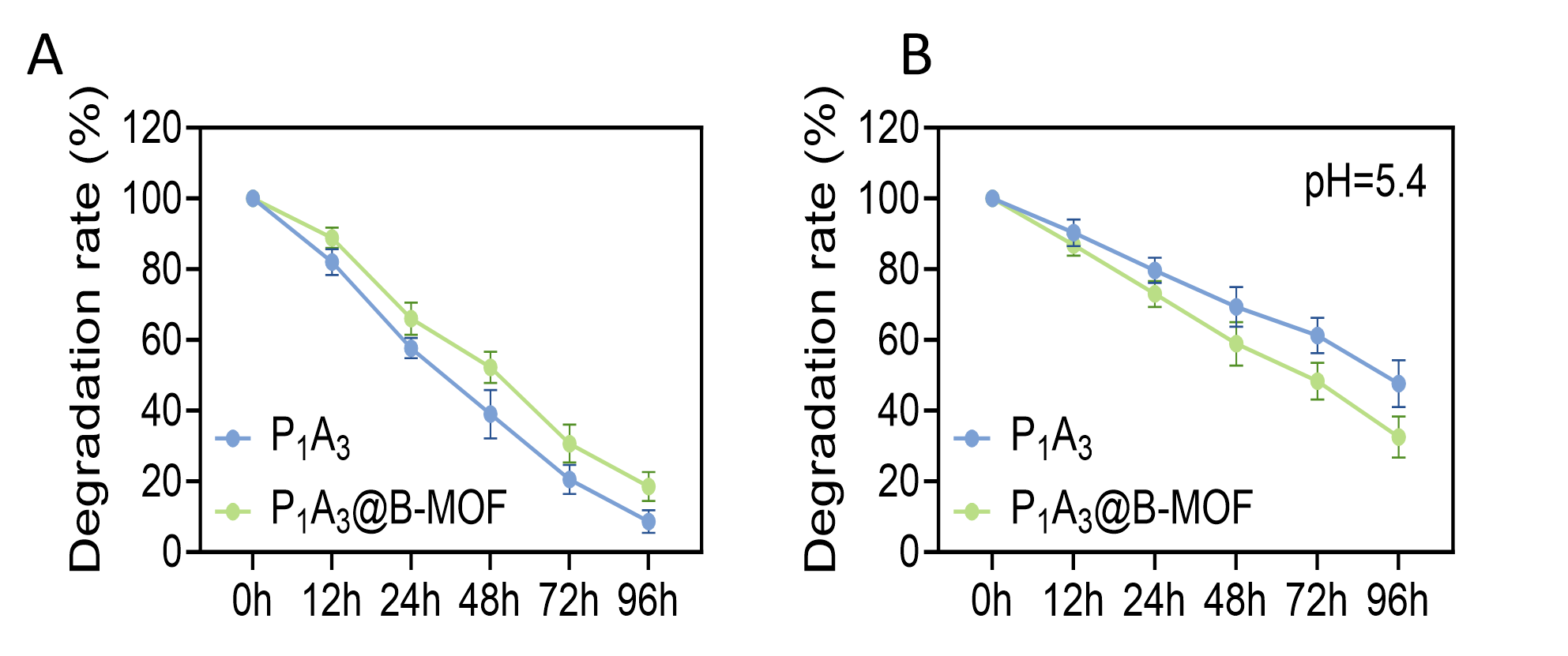


**Figure S2. In vitro degradation experiment.** (A) Degradation of sponges in collagenase solution. (B) Degradation of sponges in PBS buffer solution (pH = 5.4).


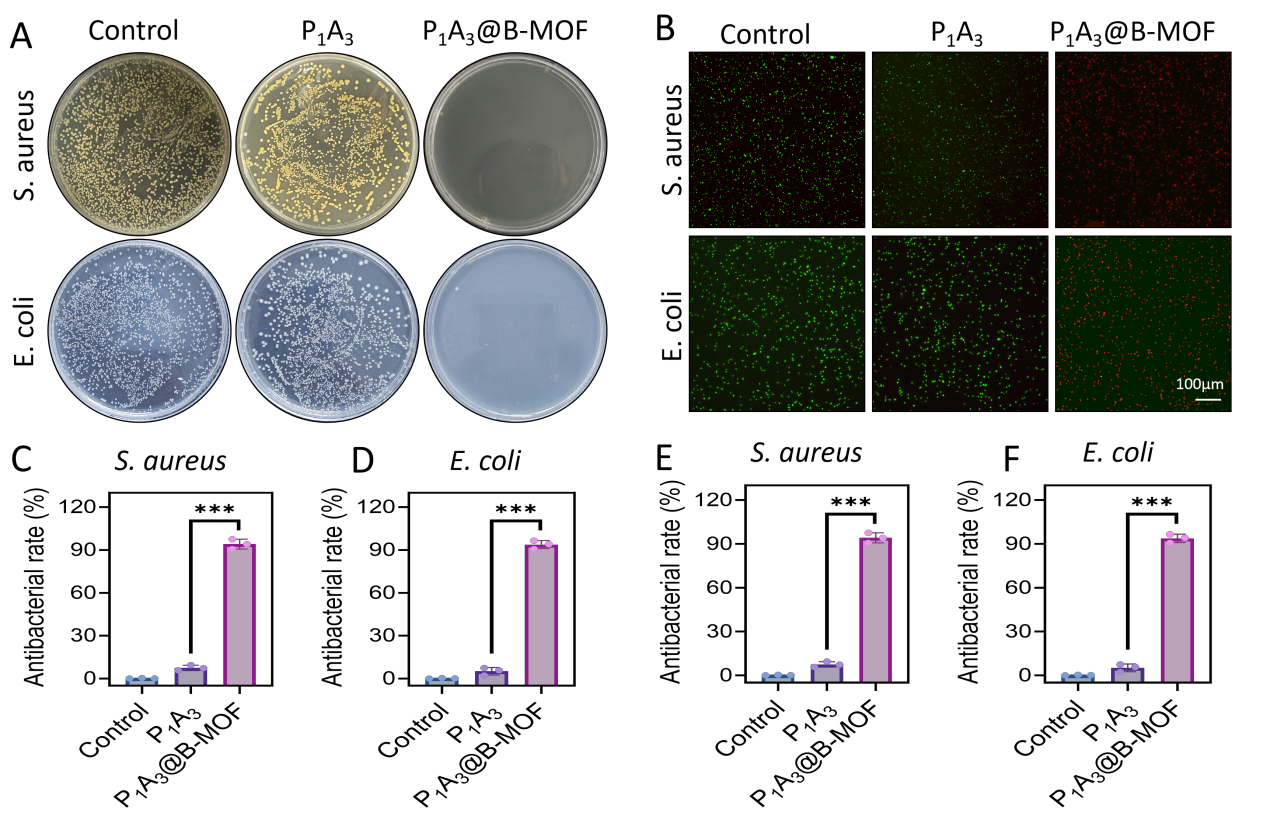


**Figure S3. Evaluation of the antibacterial properties of P_1_A_3_@B-MOF sponges.** (A) Images of agar plate counting assays for *E. coli* and *S. aureus*. (B) Fluorescence images of live and dead bacteria. (C) Antibacterial rate against *S. aureus* (plate counting). (D) Antibacterial rate against *E. coli* (plate counting). (E) Relative antibacterial rate against *S. aureus* (Live/Dead staining). (F) Relative antibacterial rate against *E. coli* (Live/Dead staining).
